# Inflammaging and Brain Aging

**DOI:** 10.3390/ijms251910535

**Published:** 2024-09-30

**Authors:** Maria Carolina Jurcau, Anamaria Jurcau, Alexander Cristian, Vlad Octavian Hogea, Razvan Gabriel Diaconu, Vharoon Sharma Nunkoo

**Affiliations:** 1 Faculty of Medicine and Pharmacy, University of Oradea, 410087 Oradea, Romania; 2 Department of Psycho-Neurosciences and Rehabilitation, University of Oradea, 410087 Oradea, Romania; 3 Emergency Clinical County Hospital Bihor, Neurology Ward, 410169 Oradea, Romania

**Keywords:** cellular senescence, neuroinflammation, senescence-associated secretory phenotype, astrocytes, microglia, immunosenescence

## Abstract

Progress made by the medical community in increasing lifespans comes with the costs of increasing the incidence and prevalence of age-related diseases, neurodegenerative ones included. Aging is associated with a series of morphological changes at the tissue and cellular levels in the brain, as well as impairments in signaling pathways and gene transcription, which lead to synaptic dysfunction and cognitive decline. Although we are not able to pinpoint the exact differences between healthy aging and neurodegeneration, research increasingly highlights the involvement of neuroinflammation and chronic systemic inflammation (inflammaging) in the development of age-associated impairments via a series of pathogenic cascades, triggered by dysfunctions of the circadian clock, gut dysbiosis, immunosenescence, or impaired cholinergic signaling. In addition, gender differences in the susceptibility and course of neurodegeneration that appear to be mediated by glial cells emphasize the need for future research in this area and an individualized therapeutic approach. Although rejuvenation research is still in its very early infancy, accumulated knowledge on the various signaling pathways involved in promoting cellular senescence opens the perspective of interfering with these pathways and preventing or delaying senescence.

## 1. Introduction

One of the medical community’s main achievements in the past century is the prolongation of lifespans [1]. Estimates by the World Health Organization (WHO) indicate that 1.4 billion people will be older than 60 by 2030, and the figure will double by 2050 [2]. However, this increase in life expectancy is accompanied by increasing numbers of elderly people living with disabilities from chronic diseases, with significant variations across countries [3], which pose a huge burden on families and society, even threatening the economies of countries facing increasing expenses for medical and social services required by elderly disabled patients [4] and forcing healthcare providers to reshape their systems in order to meet the needs of these patients.

Globally, neurological diseases are the main cause of disability, and they have escalated as the second cause of death after cardiovascular diseases [5]. Aging is the main risk factor for most neurodegenerative diseases [6], and their prevalence is rising worldwide. It is estimated that, in the United States alone, the number of Americans with diagnosed Alzheimer’s disease (AD) will more than double in the next 30 years, reaching 12 million [7], while Parkinson’s disease (PD), affecting currently around 1 million Americans, has the fastest-growing prevalence and disability rate [8].

Despite extensive research, we do not yet have effective therapeutic strategies to halt disease progression in most neurodegenerative disorders. As such, unraveling the pathogenic mechanisms of neurodegeneration and, possibly, identifying strategies to prevent these conditions appear very appealing. A growing body of evidence implicates chronic inflammation, mainly neuroinflammation, in the pathogenesis of various neurodegenerative diseases [9,10,11,12].

In this review, we will discuss the differences between normal and pathological brain aging and highlight a series of pathogenetic pathways through which neuroinflammation contributes to neurodegeneration. The references reviewed in this manuscript were obtained from the PubMed and Google Scholar databases using as search criteria “Brain aging” and “molecular pathways” and “cellular pathways” and “neuroinflammation”. We referenced full-text articles, experimental studies, and meta-analyses. No limits were set.

## 2. Aging and Senescent Cells

As we age, the functional capabilities of the various organ systems progressively decline [7], leading to an increased risk of disease and death [6]. The brain is no exception, and the human brain shrinks during normal aging, both gray and white matter being reduced at a rate ranging between 0.2–0.5%/year (with increasing rates in older ages), with a compensatory enlargement of the ventricles and subarachnoid spaces [13].

Research in the past decades has greatly increased our knowledge of the molecular mechanisms underlying aging, showing that several signaling pathways are present in *C. elegans*, flies, and mammals [14] and suggesting that the biology of aging is conserved across species.

In 2013, López-Otín and coworkers identified nine systemic, cellular, and molecular characteristics of aging, known as the hallmarks of aging, as follows [15]:

- Genomic instability;
- Telomere attrition;
- Epigenetic alterations;
- Loss of proteostasis;
- Deregulated nutrient sensing;
- Mitochondrial dysfunction;
- Cellular senescence;
- Stem cell exhaustion;
- Altered intercellular communication.

Subsequently, the same group of researchers added three more characteristics of aging, namely disabled macroautophagy, chronic inflammation, and dysbiosis [16]. However, in the present review, gut dysbiosis is discussed as a trigger of chronic inflammation and cellular senescence.

Mitosis represents the process through which a eukaryotic cell divides into two daughter cells by going through the following four phases: prophase, metaphase, anaphase, and telophase. After being formed, the cells go through an interphase period with three stages: G1, when the proteins required for DNA replication are synthesized; the S phase, with the replication of nuclear DNA; and G2, when the synthesis of proteins responsible for cell division occurs. Cells can also be found in the G0 stage, when they have withdrawn from the cell cycle [17].

By culturing diploid human fibroblast cells, Hayflick and Moorehead showed that the number of cell divisions is finite (consisting of 50–80 divisions, also known as the “Hayflick limit”) [18], after which cells enter a state of replicative senescence [19] caused by the shortening of telomeres [20], namely the nucleotide sequences of TTAGGG located at the end of chromosomes, which are not completely replicated by DNA polymerases during DNA replication and which are progressively lost with each cell division [21]. The uncapped telomeres and double-strand DNA breaks activate the DNA damage response (DDR) that stabilizes p53 through posttranslational phosphorylation via ATM (ataxia-teleangiectasia, mutated) and ATR (ATM and Rad3-related) serine/threonine protein kinases [22] or by blocking the degradation of p53 via p14^ARF^ (a tumor suppressor)-mediated inhibition of the MDM2 (mouse double minute 2) ubiquitin ligase [23]. After p53 stabilization, the transcription of the cyclin-dependent kinase (CDK) inhibitor p21 leads to the initial arrest of the cell cycle, followed by permanent arrest controlled via p16^INK4A^ transcriptional upregulation through p38 and ERK (extracellular-regulated kinase) signaling [24]. The inhibition of CDK4 and CDK6 by p16^INK4A^ causes RB (retinoblastoma protein) hypophosphorylation and the permanent blockage of S-phase entry [25].

Cellular senescence is a homeostatic response that prevents the propagation of damaged cells [26]. While cellular senescence exerts a series of physiological roles during development, it significantly contributes to an age-related loss of function later in life. However, other conditions, such as DNA damage, oxidative stress, chromatin disruption, stalled DNA replication, or the loss of tumor suppressors, can also induce cellular senescence [19,27,28]. Senescent cells remain metabolically active, and although they can be recognized and cleared via the immune system, they tend to accumulate over time across all species and contribute to age-related pathologies [26].

Research has highlighted a series of key molecular features of senescent cells:

1. They exhibit permanent cell cycle arrest caused by the increased expression of p53 [29].
2. They resist apoptosis, a resistance conferred via the accumulation of p53 and the subsequent expression of the Bcl-2 family proteins [30].
3. Senescent cells exhibit the senescence-associated secretory phenotype (SASP) dependent on p38MAPK (p38 mitogen-activated protein kinase), NF-κB (nuclear factor kappa-light-chain-enhancer of activated B cells), NOTCH, cGAS/STING (cyclic GMP-AMP synthase/stimulator of interferon genes), and mTOR (mammalian target of rapamycin) signaling and consisting of chemokines, cytokines, metalloproteinases, and growth factors that exhibit pro-inflammatory activities and act in an autocrine and paracrine manner [31], including the release of extracellular vesicles [32].
4. Senescence is also associated with changes in cellular metabolism including the upregulation of lysosomal senescence-associated β-galactosidase (SA-β-gal); increased SA-β-gal reflects an increased number or activity of lysosomes [33].
5. The accumulation of lipofuscin is another hallmark of senescence. Lipofuscin consists of lipid degradation residues and metal cations that aggregate in the cell, together with oxidized proteins, and it cannot be degraded via lysosomes, accumulating with age [34].
6. The senescent phenotype appears to be mediated by mitochondrial dysfunction via Akt (protein kinase B), ATM, and mTORC1 (the mechanistic target of rapamycin complex) phosphorylation, which link DDR with mitochondrial biogenesis [35]. Moreover, morphological changes are seen in the mitochondria of senescent cells, which tend to accumulate due to impaired mitophagy, leading to senescence-associated mitochondrial dysfunction (SAMD) and the increased production of reactive oxygen species (ROS) [36].

Commonly used markers of cellular senescence are as follows:

1. SA-β-gal originates from the lysosomal enzyme β-galactosidase and reflects an increased number or activity of lysosomes [33]. It is measured at pH 6.0 using in situ staining with the chromogenic substrate X-gal [37].
2. p16^INK4A^, a member of the INK4A family [38].
3. p21^CIP1/WAF1/SD11^; p21 is a member of the CIP/KIP family. This family comprises p21(CIP1), p27(KIP1), and p57(KIP2), which are stoichiometric cyclin-dependent kinase inhibitors. p21 and inhibits various CDKs, mediating cell death, cell cycle arrest, and DNA repair, and it is also involved in the reprogramming of differentiated somatic cells into pluripotent stem cells [39].
4. Lipofuscin binds to a biotinylated compound derived from Sudan Black (GL-13) and can thereby be detected in fresh tissues, as well as formalin-fixed and paraffin-embedded samples or biological fluids [40]. More recently, a radiolabeled derivative of Sudan Black B has allowed for the in vivo detection of lipofuscin via positron emission tomography (PET) [41].

Senescent cells of the central nervous system (CNS) may contribute to the development and progression of neurodegenerative diseases via several mechanisms:

- Loss of function. The changes in the gene expression of senescent cells may interfere with the functions of various cell types [42], and as their number increases with time, they could functionally impair the nervous system. Moreover, as senescent cells are cleared via the immune system to a certain extent, the brain volume decreases [26].
- Aging significantly reduces neurogenesis and the regenerative capacities of the CNS [43].
- The SASP of senescent cells maintains a pro-inflammatory milieu that converts neighboring cells into senescent ones in a paracrine manner, promoting chronic inflammation [44,45]. IL-6 is a crucial driver of neuroinflammation, and together with activated microglia and other cytokines and pro-inflammatory mediators, it contributes to the state referred to as “inflammaging” [46].
- Because the functions of the CNS depend on an adequate blood supply and an intact blood–brain barrier (BBB), the age-related decline in the structure of cerebral microvasculature, endothelial cell, and pericyte senescence with the associated weakening of the BBB, as well as the senescence of the cellular components of the choroid plexus, further compromises neuronal and glial function and survival [47,48,49].

### 2.1. The Senescent Neuron

The adult human brain contains an estimated 86 billion neurons [50]. Since neurons are postmitotic cells, telomere shortening has been regarded as absent in neurons. Nonetheless, cell cycle activity has been demonstrated in about 11% of postmitotic neurons in the cortex of healthy aging brains by showing variations in the DNA content [51]. In addition, transcripts for telomerase (a ribonucleoprotein enzyme with two subunits: telomerase reverse transcriptase—TERT, and telomerase RNA—TER) are maintained, although downregulated [52], and the enzyme is involved in promoting cell survival [53]. Moreover, hippocampal TERT modulates mood behaviors and controls the proliferation of neural progenitor cells (NPCs) [54].

Other age-related changes occur in the nuclei of neurons as well. Studies of transcriptional profiling of the aging human brain and primates revealed that 4% of the genes are age-regulated, and genes coding for glutamate receptor subunits, members of the signal transduction systems mediating long-term potentiation, or synaptic and vesicle proteins [55], as well as genes related to antioxidant defense, DNA repair, mitochondrial function, calcium signaling, or vesicle-mediated protein transport, are downregulated, while genes related to stress responses and immune responses are upregulated [56]. Moreover, the impairment of nuclear pores contributes to alterations in the configuration of nuclei and cytoplasm [57].

Mitochondrial alterations and dysfunction, with their consequences on energy production, calcium, and ROS signaling, are other features of senescent cells. Aging is associated with excessive mitochondrial fragmentation in the CA1 hippocampal region [58], mitochondrial enlargement in the frontal cortex [59], a decrease in respiratory-chain enzymatic complexes [9], and altered mitochondrial dynamics [60], which may ultimately ignite apoptosis.

The increase in ROS produced via dysfunctional mitochondria, xanthine oxidase, NADPH oxidase, nitric oxide synthase, peroxidases, lipoxygenases, cyclooxygenase, and endoplasmic reticulum [53] leads to DNA damage and the accumulation of oxidatively damaged proteins, lipids, and RNA [61]. DNA damage activates the p38 MAPK signaling pathway, leading to pro-inflammatory cytokine production and SA-β-gal activity via the expression of p21^WAF1/CIP1^ [62].

All of these changes at the molecular level are associated with morphological and functional changes to aging neurons. Phenotypically, neurons present pigmented accumulations, including lipofuscin, neuromelanin, and Marinesco bodies, most obvious in the dopaminergic neurons of the substantia nigra and the norepinephrine-producing neurons of the locus coeruleus [63]. Neuromelanin synthesis is driven by iron-dependent oxidation of excess cytosolic dopamine or L-DOPA [64], while Marinesco bodies, spherical eosinophilic nuclear aggregates, contain proteins [65]. Axons lose their myelin sheaths, and the myelin also shows a decrease in the water fraction [66],. These alterations, together with a deficient function of aged oligodendrocytes, lead to longer nodal and paranodal spaces [59]. The changes in spine shape and size suggest marked changes to synaptic plasticity as well [63]. The number of dendritic spines, mainly of the thin spines, decreases with age in the cortex, hippocampus, and subcortical regions [67], changes that are possibly related to cognitive decline. Microtubules are likely subject to mechanical stress, and they undergo acetylation as a protective measure, but acetylation reduces mitochondrial dynamics and alters axonal transport [68], which, together with reduced ATP availability as a consequence of mitochondrial dysfunction, act synergistically to further slow vesicle trafficking and synaptic transmission.

Signal transmission relies on a large transient sodium influx and a subsequent potassium efflux mediated via Ca^2+^-dependent potassium currents, leading to membrane afterhyperpolarization. Aged hippocampal neurons show upregulated synaptic L-type Ca^2+^ channels resulting in impaired Ca^2+^ homeostasis [62] and exhibit increased afterhyperpolarization, which interferes with the membrane’s ability to reach the action potential threshold and results in reduced firing frequency [69]. Moreover, certain neuromediator receptor subtypes, such as nicotinic acetylcholine receptor subtypes [70], dopaminergic receptors (D1, D2, and D3), and glutamate NMDA receptors, decrease with age [71].

Neural progenitor cells also exhibit features of senescence, with telomere shortening and increased ROS production [72].

In addition, with aging and age-associated metabolic dysfunctions such as obesity or peripheral insulin resistance, neurons lose their sensitivity to insulin as well [73]. Brain insulin resistance induces molecular, functional, and morphological changes characteristic of neuronal senescence [74] via increasing p25 and activating CDK5 and GSK3β (glycogen synthase kinase-3 beta) [75].

### 2.2. Astrocytic Senescence

First recognized by Santiago Ramon y Cajal at the end of the 19th century [76], astrocytes were initially thought to simply act as a glue for neurons. Comprising 20–40% of the total glial cell population [77], today, the complex functions of astrocytes are recognized and consist of regulation of embryonic and adult neurogenesis [78], glycogen synthesis, the supply of energy substrates to neurons [79], the clearance of ROS [80], the control of brain homeostasis [81], and the pruning of synapses and removal of cellular debris in cooperation with microglia [82]. They also participate in synapse transmission: while neurons take up mainly inhibitory neurotransmitters, such as gamma-aminobutyric acid (GABA), astrocytes remove and metabolize excitatory neurotransmitters such as glutamate [83], and they are major sources of extracellular matrix proteins, neurotrophic factors, and cell-adhesion molecules in the brain [84]. Moreover, via the secretion of thrombospondins and TGFβ (transforming growth factor β), they regulate synaptogenesis and the maturation of neuronal circuits [84]. Astrocytes also participate in the formation of the blood–brain barrier (BBB) and “match” cerebral blood flow with neuronal activity through the neurovascular unit (NVU) [85]. Finally, together with microglia, astrocytes participate in the immune response of the CNS [86] and, via the secretion of chemokines and cytokines, they regulate the traffic of immune cells into the CNS [87].

In response to various stressors such as DNA damage, mitochondrial dysfunction, oxidative stress, proteotoxic stress, or disrupted nutrient signaling [88], astrocytes initiate a senescence program partly dependent on p53, with increased expression of p16^INK4A^, p21^WAF1^, and CIP/KIP (CDK interacting protein/kinase inhibitory protein), which leads to cell cycle arrest independent of telomere shortening [89], referred to as stress-induced premature senescence (SIPS).

As a result of sublethal injury-induced SIPS, astrocytes alter their transcription of various genes, with a reduced expression of excitatory amino acid transporters (EAAT1 EAAT2) [90], potassium transporter Kir4, and water transporter aquaporin 4 (AQP4), thereby contributing to neuronal excitotoxicity [91]. In addition, the activity of glutamine synthase is very sensitive to oxidative stress, so available metabolic substrates to neurons will be reduced in the presence of astrosenescence [92]. The senescence-associated calcium dyshomeostasis, together with the upregulation of Ca^2+^-signaling mediators such as L-type voltage-sensitive Ca^2+^ channels, endoplasmic reticulum (ER) Ca^2+^-release channels, or Ca^2+^-binding proteins, leads to an increase in cytosolic calcium that, together with HMGB1 (high-mobility group B), modulates the activity of several transcription factors such as NF-κB, of peroxisome proliferator-activated receptors (PPARs), modulates the JAK/STAT pathway, and activates calcineurin, leading to the upregulation of key mediators of inflammation such as tumor necrosis factor α (TNF-α), interleukins (IL-6, IL-1β), or cyclooxygenase 2 (COX2) [93], chemokines such as CCL2, and matrix metalloproteinases (MMP3 and MMP9), collectively referred to as the senescence-associated secretory phenotype (SASP) [75,94].

Morphologically, senescent astrocytes exhibit enlarged nuclei and alterations in the integrity of the nuclear envelope caused by the downregulation of lamin B1 and other nuclear lamin proteins [95], with chromatin alterations and the formation of senescence-associated heterochromatic foci [96], hypertrophic processes, and an increased number of mitochondria but with altered membrane potential [97], and an increased number of lysosomes and upregulated lysosomal enzymes, including SA-β-gal [98]. In response to injury or aging, astrocytes also increase the expression of glial fibrillary acidic protein (GFAP), a cytoskeletal protein, as well as vimentin, another intermediate filament protein [62].

Due to all the aforementioned changes, senescent astrocytes convert from cells, providing trophic support to neurons to neurotoxic cells, as shown via the experimental co-culturing of neurons with senescent astrocytes [99].

**Figure 1:**
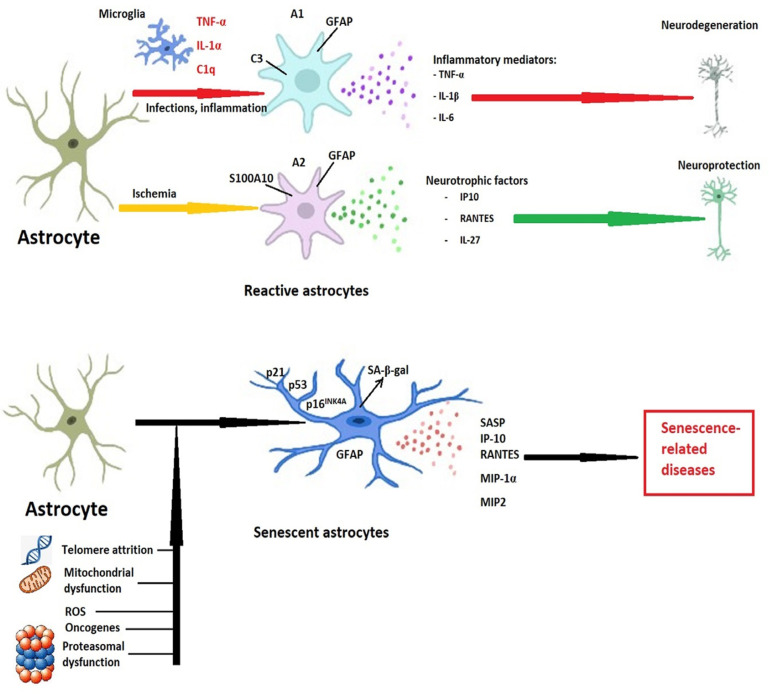
Differences between reactive and senescent astrocytes. Following infections or inflammation, the pro-inflammatory mediators produced mainly by microglia convert astrocytes to the pro-inflammatory (A1) phenotype, characterized by GFAP (glial fibrillary acidic protein) and complement component C3 secretion. These cells, in turn, secrete pro-inflammatory cytokines and lead to neurodegeneration. Although the cytotoxic phenotypes prevail in the early stages of cerebral ischemia, in time, astrocytes polarize toward the neuroprotective A2 phenotype, and express S100A10 (S100 calcium-binding protein A10) and GFAP. The neurotrophic factors and anti-inflammatory mediators produced by A2 astrocytes act as neuroprotectants and promote tissue regeneration. Telomere attrition, mitochondrial dysfunction, proteasomal insufficiency, oxidative stress, or the expression of oncogenes promote astrocytic senescence. This astrocytic state is characterized by cell cycle and proliferation arrest, increases in p16^INK4A^, p21, p53, and by secretion of senescence-associated beta-galactosidase (SA-β-gal). Moreover, a diversity of proteases, chemokines, and cytokines (senescence-associated secretory phenotype—SASP), could lead to the onset and progression of age-related diseases of the central nervous system. Abbreviations: IP-10—interferon-γ-inducible protein 10, or CXCL10; IL—interleukin; RANTES—chemokine ligand 5, or CCL5; MIP—macrophage inflammatory protein; TNF-α—tumor necrosis factor-α.

Although the secretory phenotype of senescent astrocytes resembles that of A1 reactive astrocytes polarized as a result of infections or structural lesions, several differences exist, and caution must be exerted when assessing astrocytic phenotype, as shown in Table 1 and Figure 1. Moreover, the phenotypic variations of astrocytes go well beyond the classical separation into pro-inflammatory A1 and anti-inflammatory A2 phenotypes [100].

### 2.3. Oligodendrocyte Senescence

The main function of the terminally differentiated cells of the oligodendrocyte lineage derived from oligodendrocyte progenitor cells (OPCs), also known as neuron-glial antigen 2 (NG2)-positive glia, in the CNS is to myelinate the axons of neurons. Although this process occurs at increased rates during the first two years of life, it continues to contribute to a volume increase in the white matter until midlife [104]. However, other roles are emerging, such as roles in neuronal–glial signaling, electrical activity, phagocytosis [105], and stem cell-like behavior [106]. The mature oligodendrocytes can thicken the existing myelin sheaths or lengthen the internodes, subserving myelin plasticity [107]. Myelin, aside from increasing the conduction velocity of nerve impulses, also provides metabolic support for axons by supplying lactate and pyruvate via monocarboxylate transporters [108].

Due to their high metabolic demands, oligodendrocytes are vulnerable to oxidative stress, and ROS induce DNA damage (mainly DNA double-strand breaks) that activate the p53/p16 senescent pathway [19], leading to a reduction in the myelinating capabilities. Oligodendrocyte-specific genes *MBP* (myelin basic protein) and *LINGO-1* (leucine-rich repeat and Ig-like domain-containing Nogo receptor-interacting protein 1) are downregulated with increasing age across all brain regions [109]. While the reduction in MBP is concurrent with the reduction in myelin content, LINGO-1 is a negative regulator of myelination, but it promotes the remodeling of actin filaments [110] necessary for myelination. In addition, the expression of receptors and ion channels, such as NMDA and kainate glutamate receptors, or voltage-gated sodium and potassium channels, declines with age [111]. Age-associated mitochondrial dysfunction not only reduces ATP levels but also contributes to ROS production. ROS induce the lipid peroxidation of myelin lipids, which, together with the dysfunctional cellular homeostasis and impaired membrane integrity, contributes to the altered structure of the myelin membranes [104].

Extrinsic factors, such as inflammatory cytokines released via microglia, induce nitric oxide production in oligodendrocytes, leading to hypomyelination and cell death [112]. In addition, senescent astrocytes fail to supply the necessary amounts of cholesterol for myelin synthesis to oligodendrocytes [113]. Factors released from axons, namely neuregulin-1 or neuronal adhesion molecule L1, also influence the myelination process. For example, neuronal adhesion molecule L1 activates Fyn kinase and promotes the transcription of mRNAs transported from the cell body [114].

Oligodendrocyte progenitor cells constitute about 3–10% of glial cells [115], and although they do not undergo replicative senescence, there is in vitro evidence that esophageal cancer-related gene 4 (Ecrg4), with increased expression in aged mouse brains, may cause them to enter a senescence-like state with an increased expression of SA-β-Gal and failure in their essential function of mediating remyelination via differentiation into myelinating oligodendrocytes [26]. In addition, increases in p21, p16, and SA-β-Gal have been reported in these progenitor cells [116], as opposed to mature oligodendrocytes, which are more resistant to senescent modifications, but in which senescence may occur through a p16-independent mechanism [75].

The loss of myelin leads to nerve dysfunction through secondary axonal changes such as paranode reorganization, in which the loss of clusters of ion channels at the nodes of Ranvier disrupts saltatory conduction [117]. Moreover, age-associated myelin fragmentation leaves the subjacent axon vulnerable to oxidative damage. Reduced nerve function due to myelin degeneration, in turn, may cause a positive feedback loop of reduced myelin maintenance or re-myelination [118].

### 2.4. Microglial Senescence

Microglia are the resident innate immune cells of the CNS. They have a mesodermal origin, and, together with endothelial cells and pericytes, they are the main non-neuroectodermal cells of the brain. While the age-associated alterations of microglia are described in the following section, age-induced modifications of endothelial cells and pericytes are described in Section 2.6.

In 1899, Franz Nissl described glial cells with phagocytic, migratory, and proliferative properties of mesodermal origin and termed them “rod cells” (Stäbchenzellen) [119]. Primitive myeloid precursors arise from the yolk sac following the expression of runt-related transcription factor 1 (RUNX1) and macrophage colony-stimulating factor 1 receptor (CSF1R) during development, reach the embryonic head through the blood flow, and migrate into the developing brain by using matrix metalloproteinases [120]. After the completion of the developmental process, the self-renewal of microglia maintains the population of immune cells of the CNS.

In the resting state, microglia have commonly fixed somata with motile filopodia-like processes that enable the cells to carry out immune surveillance throughout the parenchyma [121]. Every few hours, the entire cerebral parenchyma is sampled by homeostatic microglia [122]. Resting microglia are characterized by a low expression of CD68, CD22 and CX3CL1, and the neuronal plasma membrane marker CD200, as well as the expression of transmembrane protein 119 (TMEM119), Sal-like protein 1 (SALL1), TGFβ1 and TGFβ receptor 1, or sialic acid-binding immunoglobulin-like lectin H (Siglec-H), which are different transcriptomics than those of CNS monocytes or CNS-associated macrophages [123,124]. Microglia are maintained in the resting state via the interaction of specific microglial receptors with neuronal neurotrophins and neurotransmitters [125]. In addition, resting microglia downregulate MHC-I and MHC-II. Their distribution is subject to variations, being highly concentrated in the gray matter of the hippocampus, basal ganglia, substantia nigra, and olfactory cortex and being less abundant in the cerebellum and brainstem [126]. They also exhibit various spatial phenotypes: in areas lacking BBB, they are rather amoeboid; in the proximity of fiber tracts, they are mainly longitudinal branched cells, while near the neuropil, microglia appear as ramified cells [127,128].

Aside from the immune function, microglia also shape neuroplasticity [129] and facilitate learning. Suppressing microglia led to a decline in learning-associated synaptogenesis [121], and research has shown that the BDNF released via microglia increases the expression of tropomyosin-related kinase receptor B (TrkB) in neurons and leads to synaptogenesis [130]. Moreover, microglia promote neurogenesis from neural progenitor cells located in the subventricular zone and the dentate gyrus of the hippocampus [131].

During the constant surveillance of the CNS environment, microglia recognize both foreign (bacterial or viral) molecules and endogenous proteins or DNA and RNA released from damaged cells via runt-related transcription factor 1 (belonging to the PRRs) located on the microglial membrane. Most of these PRRs are toll-like receptors (TLRs), triggering receptors expressed on myeloid cells (TREMs), or nucleotide-binding oligomerization domain (NOD)-like receptors (NLRs) [132]. The interaction of the ligands with these receptors triggers a series of signaling pathways that lead to an upregulated CD68 profile and the production of pro-inflammatory cytokines [interleukin (IL)-1β, IL-6, IL-12, IL-18, tumor necrosis factor (TNF)-α, matrix metalloproteinases MMP-9 and MMP-12, interferon γ (IFNγ) and cyclooxygenase-2 (COX2)], chemokines [C-C motif chemokine ligand 1 (CCL1), CCL5, and C-X-C motif ligand 1 (CXCL1)], or small-molecule messengers (prostaglandins, nitric oxide, and ROS) to mediate the neuroinflammatory response [133] and promote the phagocytosis of damaged cells or protein aggregates via the activated microglia [132]. For an efficient response, microglia cooperate with astrocytes and capillary endothelial cells, and they promote the infiltration of peripheral immune cells through a “leaky” BBB [134]. ATP released from injured brain cells induces an astrocyte-derived ATP gradient that acts on the microglial purinergic receptor P2RY12 and results in microglial migration and activation [135]. The pro-inflammatory cytokines (IL-1α and TNF-α) and C1q produced via activated microglia induce the so-called “A1” or neurotoxic reactive astrocyte phenotype [136].

In situations of systemic inflammation, CCL5 released from endothelial cells triggers microglial cells to interact with the endothelium and promote the formation of tight junctions in an attempt to maintain BBB integrity. Nonetheless, sustained inflammation leads to microglial activation and polarization towards the M1 phenotype, with a resultant weakening of the BBB via microglial engulfment of the astrocytic end feet [137]. Subsequently, systemic adaptive immune cells, such as lymphocytes and macrophages, can infiltrate the CNS and perpetuate the neuroinflammatory state by interacting with glial cells, or they can modulate the immune response by releasing various cytokines [138]. For example, microglia depletion and transcriptomic analysis in mice after an intravenous administration of bone marrow suggests that peripheral macrophages can replace microglia [139], opening the possibility of targeting microglia-mediated neuroinflammation by engrafting macrophages to the CNS [140]. Anti-inflammatory cytokines, such as IL-1 receptor antagonist, IL-4, IL-10, or IL-11, partly resulting from the interaction between microglia and monocyte-derived macrophages, prevent excessive inflammation and promote tissue repair by favoring the shifting of the microglia toward the anti-inflammatory M2 phenotype [141], characterized by the expression of IL-4, IL-10, IL-13, BDNF, and TGF-β [142]. However, the classical M1–M2 dichotomy of the microglial phenotype is oversimplified, and research has shown that the microglial transcriptome differs in various brain insults, such as neurodegeneration, ischemia, or infection [121]. Table 2 summarizes the protective and detrimental microglial signaling pathways.

Decreased arterial blood flow and reductions in glucose catabolism associated with aging lead to a sustained activation of microglia that maintains a chronic neuroinflammatory state [154]. A transcriptional evaluation of neurodegenerative-phenotype microglia has shown the downregulation of a series of genes, such as *Tmem 119*, *P2ry12*, myocyte enhancer factor 2A (*Mef2a*), or spalt-like transcription factor 1 (*Sall1*), via a decline in the important transcription factor TGF-β [121]. These alterations impair their lipid metabolism and phagocytic ability, and they can even lead to morphologic changes such as “dark microglia” (named after their appearance under an electron microscope), characteristically found in aged brain tissues or specimens from Alzheimer’s disease (AD) models [155]. The morphologic change could reflect cellular shrinkage, but it is also associated with features of oxidative stress, such as the dilation of the Golgi apparatus, the condensation of the nucleoplasm, and alterations in mitochondrial morphology and integrity [156], also being highly ramified and encircling synaptic elements [157]. This microglial phenotype appears to prevail in regions adjacent to large blood vessels, suggesting that they appear as an attempt to preserve BBB integrity [157,158].

However, aged microglia, together with a weakened BBB, lead to an increase in circulating IL-6 [159,160], TNF-α [161], intercellular adhesion molecule-1 (ICAM-1), the tissue inhibitor of metalloproteinases 1 (TIMP-1), and glial fibrillary acidic protein (GFAP), which are associated with sarcopenia and physical frailty [162]. Moreover, a rise in the microglial load of the brain tissue correlates with a reduction in the activation of neural progenitor cells [163].

**Figure 2:**
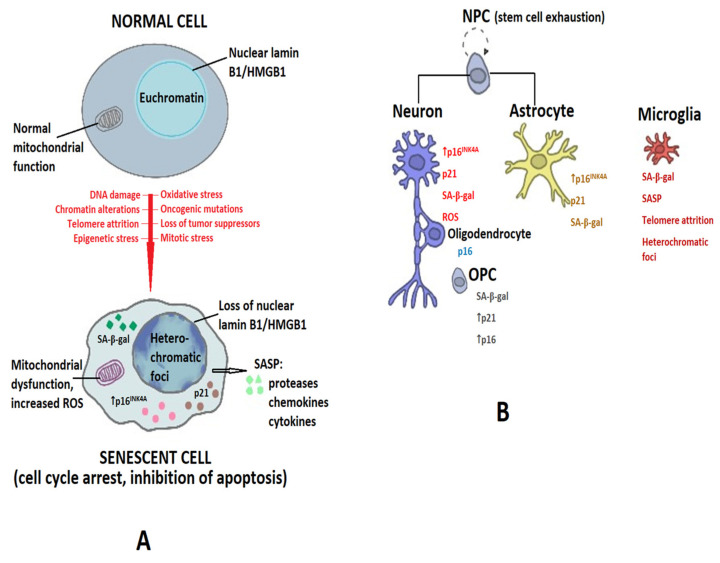
Senescence-associated phenotypes of brain cells. (**A**) Diverse stressors (DNA damage, chromatin alterations, telomere attrition, epigenetic stress, oxidative stress, oncogenic mutations, loss of tumor suppressors, or mitotic stress) promote the conversion of normal cells into senescent cells (shown by red arrow). Senescent cells are characterized by cell-cycle arrest and resistance to apoptosis, and by the presence of heterochromatic foci, the increased production of reactive oxygen species (ROS), and increases in senescence-associated β-galactosidase (SA-β-gal), p16^INK4A^ (shown by the short black arrow) and p21, and by the release of a series of proteases, chemokines, and cytokines collectively known as senescence-associated secretory phenotype (SASP), as shown by the large black arrow. (**B**) Markers of senescence in the main cell types in the CNS. Abbreviations: NPC—neural progenitor cell; OPC—oligodendrocyte progenitor cell.

Figure 2 summarizes the senescence-associated phenotypes and markers in the main cells of the CNS.

### 2.5. Morphological Changes to the Brain with Aging

Age is associated with alterations in brain morphology (shape and anatomy), with a significant impact on memory, motor performance, and learning abilities [13,164]. The initial changes occur at the cellular level due to ischemia and the slowing of metabolic activity [62], followed by tissue- and organ-level changes [165].

Studies have shown that the volume and weight of the brain decrease by 5% per decade after the age of 40 [166], but they are unevenly distributed across the different brain areas: while the frontal lobe decreases by 12% and the temporal lobe by 9%, the occipital and parietal lobe exhibit insignificant changes [167].

Commonly used tools to assess these changes are structural magnetic resonance imaging (MRI), functional MRI, and positron emission tomography (PET). T1-weighted MRI allows for the evaluation of volume and cortical thickness, while T2-weighted fluid-attenuated inversion recovery (FLAIR) allows for the characterization of white-matter abnormalities. The analysis of white-matter axon fibers, tissue anisotropy, and the direction of myelin water movement in extra- and intracellular white matter can be performed with diffusion tensor imaging (DTI), and functional MRI detects neuronal activity during a resting state or task performance [168]. PET enables the measurement of cerebral blood flow, metabolism, and regional chemical composition, and it can detect targeted disease biomarkers [169].

Research has consistently shown a decline in the volume of gray matter with aging at rates that may vary with gender, being about 0.4%/year in men and 0.29%/year in women [170], but with regional variations, striking mainly the hippocampus and dentate gyrus [171], while the entorhinal cortex is, surprisingly, one of the most resistant areas to aging [172]. However, the number of cells decreases by only about 2–4%, while most of the volume loss is due to cell shrinkage and the degeneration of the dendritic network [173,174].

The white matter volume decreases more rapidly, not only at estimated yearly rates around 0.77–0.88% after the age of 70 [175] but also with regional variations, being more prominent in the frontal lobe [176]. The underlying mechanisms relate to alterations in axonal architecture (shortening of the axons by 10% per decade, increase in extracellular water, and accumulation of harmful plasma proteins) [177], demyelination [178], and the accumulation of white-matter hyperintensities [179].

The width and depth of the cortical sulci also increase with age, mainly in the frontal lobe [13]. The loss of brain volume is associated with an enlargement of the cerebral ventricles with an excessive accumulation of CSF and the compression of brain parenchyma [180] due to the impaired cerebral venous drainage [181] and the weakening of the brain–CSF barrier [182].

### 2.6. Aging and the Blood–Brain Barrier

The brain is an extensively vascularized organ with 644 km in the total length of brain vessels and 20 m^2^ of vascular surface [183]. Nonetheless, the BBB “shelters” the CNS from peripheral toxins or microorganisms, having a crucial contribution to brain homeostasis. The functional unit of the BBB is the neurovascular unit (NVU), composed of brain endothelial cells, pericytes, a basement membrane layer, and astrocytic endfeet [184]. More recently, the BBB has been divided into four barriers: (a) the vascular BBB (vBBB), at the level of the arterioles, capillary bed, and venules, where the main function is exerted by the endothelial cell; (b) the blood–cerebrospinal fluid barrier, at the level of the choroid plexus, with the ependymal cell being the main player; (c) the meningeal barrier, located at the level of the arachnoid, with the main role ascribed to the endothelial cells; and (d) the tanycytic barrier, separating circumventricular organs from areas of barriered brain, whose fundamental organ is the tanycyte [185]. All of these barriers change with aging.

Brain endothelial cells have a thick luminal glycocalyx layer, are united by tight junctions (TJ) that limit paracellular diffusion, lack fenestrations, and have selective transporter systems for both the influx and efflux of various molecules [186]. These transporters are vesicles, channels, or pores that uni- or bidirectionally transit the BBB and are either energy-dependent or energy-independent [185]. A series of these transporter systems decrease with age, such as large neutral amino acid transporters, those for the interleukin-1 family, choline, glucose, TNF-α, or enkephalins [185,187], although it is still a matter of debate whether these changes are a cause or consequence of CNS dysfunction, reflecting brain atrophy and reduced demand. Nonetheless, inhibition with normal aging of the brain-to-blood transporter of low-density lipoprotein receptor-related protein-1 (LRP-1) leads to decreased efflux of amyloid beta (Aβ) peptide and is one of the mechanisms leading to Aβ accumulation in the brain [188]. Age-related alterations of the glycocalyx of brain endothelial cells in aging humans have yet to be studied [185].

Even during normal aging, the BBB deteriorates and is characterized at the functional level by an increased permeability to serum albumin [189] and at the cellular level by dysregulation in the expression of TJs [190], altered transport systems [191], and a decrease in the pericyte coverage of cerebral blood vessels [192]. Single-cell RNA sequencing from brain endothelial cells of aged mice revealed an increase in the number of p^21^-positive cells, while transcriptomic analysis showed a downregulation of occludin and several transporter genes [193]. Moreover, in an in vitro model of senescent BBB, endothelial cells were found to express SA-β-gal, p21, decreased levels of occluding, and the extravasation of high-mobility group box protein 1 [193]. Although astrocytes undergo hypertrophy and take on a more “reactive” phenotype with aging, these changes do not affect the ability of the astrocytic end feet to maintain the BBB [194]. However, they are the main cells that secrete sonic hedgehog protein that regulates BBB permeability, and the diminished sonic hedgehog signaling in aging may contribute to age-related BBB dysfunction [195].

Pericytes are thought to primarily derive from the neural crest and mesenchymal cell lineages [196], with a subset originating from blood-borne macrophages [197]. Pericytes are embedded in the basal membrane and attached to the endothelial cells of the BBB via a “peg and socket” structure [198]. They regulate the formation of astrocytic end feet and BBB endothelium, also interfering with the transport systems [199] and transmigration of immune cells such as monocytes and lymphocytes [200]. During aging, pericytes have been shown to exhibit lipofuscin inclusions, changes in mitochondrial size, and overall changes in structure and morphology, as well as pericyte loss, with subsequent detrimental effects on BBB permeability and neurovascular regulation [201]. The transcription of the gene *ARHGAP42* declines with age [202], the protein being able to regulate blood pressure [203]. Moreover, the loss of laminin secreted by pericytes enhances BBB permeability [204]. Pericyte loss impacts the microcirculation within the brain, leading to oxidative stress in the hypoperfused cerebral areas, with ROS being able to trigger inflammation and directly leading to neuronal loss [205]. In pathological states, such as AD, Aβ oligomers signal to pericytes, leading to capillary constriction [206]. In addition, pericytes clear Aβ through receptor-mediated endocytosis, involving the low-density receptor-related protein 1 (LRP1), a function altered by age-related dysfunction and loss of pericytes [207].

The basement membrane is a 40–100 nm-thick layer of extracellular matrix on the abluminal surface of the brain endothelium synthesized by endothelial cells, astrocytes, and pericytes, consisting of a backbone of the heterotrimer laminin and the sheet-like collagen IV stabilized by cellular fibronectin and heparan sulfate proteoglycans [208]. With age, and precipitated by increases in systolic blood pressure and widened pulse pressures, a thickening of the basal membrane has been described in various studies [209]. This thickening leads to alterations in the composition of the basement membrane, with an increase in collagen IV [210], an increase in fibronectin, and the deposition of lipid droplets [211].

The glymphatic system has important contributions to the removal of waste products from the brain. Solutes that are not cleared across the vascular BBB are taken up by the CSF and conveyed into the bloodstream via the arachnoid villi or drained along the cranial nerves into the cervical lymphatics. The rate of CSF turnover decreases with age [212], and the loss of astrocytic end feet aquaporin 4 impairs the normal function of the glymphatic system [213]. Many age-related changes occur in the choroid plexus, such as a 15% reduction in the height of the epithelial cells and a 10% reduction in the length of microvilli [214], which leads to a reduction in CSF secretion [215]. Moreover, transporters and enzymes in the choroid plexus, such as aquaporin 1 and Na^+^-K^+^-ATPase, have also been shown to decline in aged rats [216]. Immune quiescence in the plexus is supported by klotho, which diminishes with age and, together with peripheral immune senescence, drives neuroinflammation [217].

Although traditionally considered an organ devoid of lymphatic vessels, the CNS has some features of lymphatic vessels in the meninges [218]. The initial lymphatic vessels have small, button-shaped junctions with discontinuous basement membranes and lack smooth muscle cells. The button junctions and anchoring filaments construct the primary lymphatic valves that permit the entry of interstitial fluid, macromolecules, and immune cells [219]. These initial lymphatic vessels drain into pre-collecting and collecting lymphatics that have tight junctions between endothelial cells and secondary intraluminal valves that prevent lymphatic backflow [220]. The meningeal lymphatic vessel flow runs countercurrent to venous flow in the superior sagittal sinus [221]. The collecting meningeal lymphatic vessels at the base of the skull then extend along the jugular vein and confluence with the peripheral collecting lymphatics [222]. The meningeal lymphatic vessels account for approximately 30–50% of the CSF outflow and drain macromolecules, antigens, immune cells, and interstitial fluid, as well as waste products to maintain homeostasis [223]. The term “glymphatic” system, first proposed by Nedergaard, drives CSF influx into the brain parenchyma along the peri-arterial space, while aquaporin-4 expressed by vascular astrocytic end feet promotes glymphatic transport and the mixing of CSF with interstitial fluid [224]. These vessels perform important functions in clearing metabolites and misfolded proteins, as well as in the trafficking of immune cells [219]. Aging is associated with a decreased ability to drain immune cells via meningeal lymphatic vessels [225] to clear misfolded proteins and waste products, as well as a thickening of the vessel walls [226].

Tanycytes in the adult brain are considered to be residual radial glial cells [227]. They occupy the floor and lateral walls of the third ventricle and are found in some circumventricular organs such as the subfornical and subcommisural organs, the pineal gland, the organum vasculosum of the lamina terminalis, the area postrema, and the median eminence [228]. Being exposed to the CSF, they have access to plasma metabolites and hormones through fenestrated capillaries [229]. Hypothalamic tanycytes play a crucial role in regulating energy uptake and expenditure. During fasting, the barrier function of tanycytes is altered through a VEGF (vascular endothelial growth factor)-A dependent mechanism to allow enhanced vascular permeability and contact between circulating metabolites and neurons of the arcuate nucleus [230]. Also, leptin, produced by adipocytes, is taken up by tanycytes and released into the CSF of the third ventricle, from which it reaches the neurons of the arcuate nucleus. However, studies in rats showed that, with age, the number of tanycytes is reduced by 30%, and the remaining cells express GFAP [231]. Moreover, aged tanycytes showed significant intracellular separations, with only fine cytoplasmic processes remaining to interlink them, which could potentially impair the integrity of the blood–brain–cerebrospinal fluid barrier [232].

### 2.7. Aging and the Immune System

The contribution of the innate immune system has long been discussed, but more recently, the role of the adaptive immune system, and mainly of T-cells, is increasingly highlighted [233].

The innate immune system comprises a series of cell types able to recognize pathogen-associated molecular patterns (PAMPs) or danger-associated molecular patterns (DAMPs), and it react in a non-specific manner [234]. The monocyte chemotactic protein 1 (MCP-1) secreted by senescent hematopoietic cells facilitates tissue infiltration with macrophages, CNS included [235]. However, due to environmental factors related to SASP, macrophages in aged brain tissue are primed with inflammatory cytokines and increase the expression of MHC-II and CD40, leading to impaired synaptic plasticity and the inhibition of long-term potentiation [236]. Neutrophils become less efficient in clearing tissue debris, having an impaired phagocytic capacity and producing more ROS [237], thereby leading to an impaired response to infection or the sterile injury of tissues. Dendritic cells, crucial for antigen presentation and for maintaining the balance between immune tolerance and aberrant immune responses, are significantly impacted by age [235]. They have a reduced ability to stimulate the proliferation of CD4^+^ and CD8^+ T-cell^s and impaired phagocytic abilities, prolonging exposure to self-antigens and promoting auto-inflammation in aged hosts. Research has also shown that they tend to accumulate in the aging brain [238].

Studies on the T-cell pool have shown that, with age, the naïve T-cell compartment decreases, and the memory T-cell compartment increases, presumably caused by the exposure of T-cells to various antigens throughout life, thymic involution, and the impaired homeostatic proliferation of naïve T-cells [239]. In the elderly, memory T-cells lose the expression of co-stimulatory molecules, such as CD28 and CD27, and display mitochondrial dysfunction, signs of DNA damage, and shortened telomeres, activating senescence-associated signaling pathways [233]. Senescent T-cells exhibit T helper (TH)1, TH9, TH17, or activated regulatory T-cell (Treg) phenotypes and increase the secretion of pro-inflammatory and cytotoxic cytokines [240], thereby driving age-associated chronic inflammation. Experimental T-cell-specific deletion of the mitochondrial transcription factor A (TFAM) in mice resulted in an extremely differentiated TH1 phenotype, premature inflammation, cognitive decline, and a reduction in lifespans by 50% [241]. Several mechanisms through which T-cells may contribute to age-related diseases have been proposed [233]:

- The sustained production of cytokines, such as interferon-γ and TNF, can activate SASP in neighboring cells, which promotes TH1 and TH17 differentiation and boosts inflammation in a feed-forward loop. In addition, the secretion of granzyme K via exhausted T-cells promotes SASP of senescent cells.
- Dysfunctional T-cells fail to clear senescent and irreversibly damaged cells.
- Senescent CD4^+^ and CD8^+^ T-cells can secrete cytotoxic granules that can directly damage cells in tissues, leading to the impairment of self-tolerance.
- T-cells can modulate gut homeostasis (detailed below).

Immune cells can enter the brain parenchyma through the meningeal lymphatic vessels and regulate important functions. Moreover, the weakened BBB characteristic of old age can enhance the influx of immune cells [242]. Tissue-resident memory T-cells have been found to populate the white matter of middle-aged healthy persons [243], together with CD4^+^CCR5-high T-cells expressing the VCAM-1 ligand VLA4, which promotes their against-flow movement and search for sites, allowing their extravasation. Moreover, following VLA4 binding to VCAM-1, these cells produce granzyme K that induces local ICAM-1 aggregation and facilitates endothelial transmigration [243].

A series of experimental studies in mice lacking T- and B-cells have highlighted the involvement of these cells in learning. Meningeal IL-4-producing T-cells maintain meningeal myeloid cells in the resting state. IL-4-deficient mice have been shown to have inflammatory myeloid cells and exhibit cognitive impairment that can be reversed by the transfer of wild-type T-cells [244]. Further, meningeal T-cells, presumably via IFN-γ secretion, regulate neuronal connectivity and social behavior; IFN-γ receptor-knockout mice have significant deficits in social interactions [245].

## 3. Neuroinflammation Pathways in Brain Aging

A growing amount of evidence points toward an important contribution of chronic inflammation to the aging of all organ systems [246,247]. Normally, an inflammatory event involves cellular and molecular events that are self-limiting, followed by a resolution phase of inflammation. The unsuccessful resolution of this inflammation leads to the sustained recruitment of inflammatory cells, a lack of clearance of cellular debris and dead cells, and the failure of macrophage switching to the anti-inflammatory and regenerative phenotype [248]. Chronic inflammation is a characteristic of aging, and it is accompanied by cellular senescence, immunosenescence, organ dysfunction, and age-related diseases, such as non-alcoholic fatty liver disease, cardiovascular diseases, pulmonary fibrosis, chronic obstructive pulmonary disease, type 2 diabetes mellitus, and other conditions. These consequences are particularly important at the level of the brain [53], given the crosstalk between the nervous system, the immune system, and the endocrine systems via a series of neurotransmitters, cytokines, and hormones [248]. A series of molecular pathways have been convincingly linked to neuroinflammation, and they act synergistically to contribute to brain aging, as well as to several neurodegenerative diseases.

### 3.1. Nuclear Factor-κB in Neuroinflammation

First described as a B-lymphocyte cell-specific transcription factor that binds to the κB site in the immunoglobulin kappa-light-chain-enhancer in B cells [249], nuclear factor kappa enhancer binding protein (NF-κB) is present in all cell types [250].

The family of NF-κB is composed of structural homologues that include NF-κB1 (p50), NF-κB2 (p52), RelA (p65), RelB, and c-Rel [251]. In the cytoplasm, NF-κB is maintained in an inactive form by being bound to inhibitory proteins such as p105, p100, and IκB α, β, γ, or other binding proteins [252]. Once detached from these inhibitors, NF-κB proteins bind to κB sites, which are specific sequences of DNA, and promote the transcription of various genes. However, the final result of NF-κB activation depends on the cell type: while its activation in glial cells leads to neuroinflammation and apoptosis, in neurons, it rather promotes cell survival and neuronal plasticity [253].

To initiate the immune response, the NF-κB pathway is initiated via toll-like receptors (TLRs) on microglia, which contain an extracellular leucine-rich repeat domain (LRR) involved in pathogen recognition, a Toll/IL-1 receptor (TIR) domain in the cytoplasmic region involved in the signaling pathway, and a myeloid differentiating factor 88 (MyD88), which is an adapter protein, also activating a series of signal transduction pathways [251]. Ligand (lipopolysaccharide, TNF-α, IL-1β, etc.) binding to TLRs ignites the intracellular kinase signaling cascades, in which a ternary IκB kinase (IKK) complex [consisting of two catalytic subunits, IKKα and IKKβ, and a regulatory subunit called the inhibitor of κB kinase gamma (IKKγ) or NF-κB essential modifier (NEMO)] induces IκBα protein phosphorylation and ubiquitination, disrupts the interaction between IκBα and NF-κB, and results in nuclear translocation of NF-κB and transcription of specific genes.

### 3.2. TNF-α and Its Signaling Pathways

TNF-α is an inflammatory cytokine that binds to receptors containing a homologous cytoplasmic sequence identifying an intracellular death domain, such as tumor necrosis factor receptor 1 (TNFR1) (p55) or TNFR2 (p75) and CD95 (APO-1/Fas) with their corresponding death ligands, TNF-α, and the type II transmembrane protein, FasL. While TNFR1 is expressed in all cell types and preferentially binds a soluble protein fragment of TNF, TNFR2 is expressed mainly in cells of the immune system and endothelial cells and is activated by the transmembrane form of TNF [11]. TNFR1 contains an intracellular TNF-receptor-associated death domain (TRADD), which, upon TNF binding, interacts with FAS-associated death domain (FADD) and activates caspase 8 and caspase 3, leading to apoptosis, while TNFR2 interacts with TNF receptor-associated factors (TRAF1, TRAF2, and TRAF3), which in turn interact with the cellular inhibitor of apoptosis proteins 1 and 2 (CIAP1/2), NF-κB-inducing kinase (NIK), and phosphoinositide 3 kinase (PI3K) to promote cell survival via complex pathways [11].

### 3.3. ROS-Induced Neuroinflammatory Pathways

Already in the 1950s, Harman suggested that ROS cause oxidative damage in cellular macromolecules, leading to decreased physiological function associated with aging [254].

In neurons, the types of ROS include superoxide anion produced via the mitochondrial respiratory chain and by different oxidases, hydroxyl radical generated via the hydrogen peroxide reaction with Cu^+^ or Fe^2+^, and nitric oxide (NO) produced in response to increased intracellular levels of Ca^2+^ [255]. These molecules must be rapidly converted to non-reactive molecules via the antioxidant enzymes (glutathione, glutathione reductase, glutathione peroxidase, catalase, superoxide dismutase, and heme oxygenase-1), the transcription of which is regulated mainly via the nuclear factor erythroid 2-related factor 2 (Nrf-2) [256]. Normally, Nrf-2 is sequestered by Keap1, which promotes Nrf-2 ubiquitination and proteasomal degradation; the oxidation of cysteines in Keap1 promotes its dissociation from Nrf-2. Alternatively, Nrf-2 can be activated via phosphorylation via protein kinase C or casein kinase-2 or by interacting with MAPK [11]. Enhanced ROS generation and decreased antioxidant defenses associated with aging result in oxidative stress and oxidative damage to various molecules, especially DNA. Particularly mitochondrial DNA is vulnerable to oxidative attack due to the lack of protective histones [11]. However, it appears that cytosolic ROS is more harmful than mitochondrial ROS [257].

In addition, excess nitric oxide (NO) generated both in mitochondria during the conversion of L-arginine to L-citrulline and from the induction of inducible nitric oxide synthase via activated microglia [258] interferes with the homeostatic function of NO, decreases intracellular glutathione levels, and promotes nitrosative and oxidative damage to proteins, lipids, and nucleic acids [258].

### 3.4. RAGE Signaling Pathway

The receptor for advanced glycation end products (RAGE), expressed by microglia, neurons, astrocytes, endothelial cells, and pericytes, belongs to the group of PRRs that interacts with PAMPs and DAMPs to induce the innate immune response [259]. The receptor can be activated not only via advanced glycation end products (AGEs) but also via advanced oxidation protein products, advanced lipoxidation end products, heat shock protein 70 (HSP70), high-mobility group box 1 protein (HMGB1, or amphoterin), complement components C3a and C1q, or members of the S100/calgranulin protein family [258,260]. In contrast to the large number of ligands able to interact with its extracellular domain, the intracellular domain interacts mainly with actin-regulating protein—diaphanous 1 (DIAPH1, formerly named mDia1) [261] and via a cascade of signaling pathways, such as the JAK/STAT, MAPK, NF-κB, and PI3K/Akt pathways, leading to the production of pro-inflammatory cytokines and ROS via microglia and astrocytes or the downregulation of homeostatic molecules with the subsequent cellular destruction and demyelination of axons [259]. However, ligand binding to RAGEs has been suggested to also induce MyD88 (myeloid differentiation primary response 88)-dependent pro-inflammatory signaling similar to TLR signaling, with Toll/interleukin-1 receptor domain-containing adapter protein (TIRAP) [262], a regulatory protein, acting as a bridge between the two pathways [258]. In aging, especially in the presence of diabetes, the levels of RAGE ligands increase, and the expression of RAGEs is upregulated, thereby strengthening the contribution of this signaling pathway to bot-systemic inflammation and neuroinflammation [263].

### 3.5. The cGAS-STING Pathway

One of the main ways by which the immune system protects against pathogens is by recognizing foreign nucleic acids. Cytosolic DNA can be sensed by cGAS (cyclic GMP-AMP synthase) [264], which activates its catalytic activity and induces the synthesis of 2′3′cyclic GMP-AMP (cGAMP) [265]. cGAMP binds to STING (stimulator of interferon genes), followed by translocation of STING from the endoplasmic reticulum to the Golgi apparatus and the recruitment of TANK binding kinase 1 (TBK1), which phosphorylates STING and interferon regulatory factor 3 (IRF3). Phosphorylated IRF3 translocates to the nucleus and promotes the production of pro-inflammatory cytokines and interferons (IFNs). Alternatively, phosphorylated STING can also activate IκB kinase (IKK), leading to the phosphorylation of the inhibitor of κB (IκB) and the release of NF-κB, the master transcription factor regulating the production of pro-inflammatory cytokines, such as IL-6 and IL-12 [9]. Further, interferon can emit a signal via the heterodimeric receptor IFNAR and the JAK/STAT pathway [266].

Aside from viral or bacterial DNA, the cGAS-STING pathway can also be activated via self-DNA released into the cytosol from the nucleus or mitochondria under conditions of cellular or mitochondrial stress [258].

The activation of the cGAS-STING pathway induces autophagy (promoting the clearance of intracellular protein accumulations in neurodegenerative diseases), potassium efflux, and the activation of the NLRP3 inflammasome, as well as pyroptosis [267]. Nonetheless, the contribution of this pathway to aging and neurodegeneration is still under research. While the knockout of cGAS in mice proved protective from Aβ pathology and cognitive impairment [268], cGMP was shown to induce signaling via TREM2 (triggering receptor expressed on myeloid cells 2), possibly leading to decreased Aβ deposition and improved cognitive abilities [269]. It has been suggested that the mild activation of microglia and astrocytes maintains neurogenesis, neurite outgrowth, and synaptic plasticity [270], while the stronger activation of glial cells leads to neurodegeneration via the activation of p53 and NF-κB [266].

In aging, dysfunctional mitochondria trigger a ROS-JNK retrograde signaling pathway, leading to cytosolic fragments of chromatin that can induce the SASP via the cGAS-STING pathway. Moreover, the expression of DNAse TREX1 is downregulated in aging, resulting in increased levels of cytoplasmic DNA [271].

### 3.6. Inflammasome Signaling

Inflammasomes are multiprotein complexes assembled in the cytosol, consisting of a sensor protein, the adapter apoptosis-associated speck-like protein (ASC), and procaspase-1 [272]. The sensor protein, belonging to the family of pattern-recognition receptors, can be either membrane-bound (TLRs or C-type lectins) or cytoplasmic receptors, such as the retinoic acid-inducible gene-1 (RIG-1)-like receptors, NOD-like receptors (NLRs), or absent in melanoma 2 (AIM2) [273]. Following the activation of the sensor protein, it oligomerizes and binds ASC containing a caspase recruitment domain (CARD), which will recruit pro-caspase-1 and transform it into active caspase-1. The latter further processes pro-IL-18 and pro-IL-1β into active cytokines [258]. Activated caspase-1 also cleaves gasdermin D, augmenting the release of pro-inflammatory cytokines and leading to plasma-membrane rupture and pyroptosis [258]. Of the numerous inflammasomes identified to date (NLRP1, NLRP2, NLRC4, and others), NLRP3 is the most studied one. A two-signal model has been suggested to underlie NLRP3 activation. The priming signal is triggered via cytokines or foreign molecules binding to TLRs and the activation of NF-κB, which increases the expression of pro-IL-1β. The second signal can be provided by ATP, pore-forming toxins, non-self-nucleic acids, ROS, and others [274].

During aging, the functional decrease in the immune system enhances the role of inflammasome signaling, with inflammasomes being assembled mainly in microglia and macrophages, and also, to a lesser degree, in dendritic cells, astrocytes, oligodendrocytes, and neurons [273]. Inflammasomes are used in cells to promote the clearance of cellular debris, accumulated damaged proteins, and senescent cells. However, DAMPs activate the PRRs and stimulate the inflammasomes, which, in turn, release pro-inflammatory cytokines that maintain chronic, low-level inflammation, driving the SASP [275]. Caspase-1, caspase-11, ASC, and gasdermin levels were found to increase in the hippocampus of aged mice [276], and transcriptomic analysis showed that about 50% of genes regulating and promoting inflammation and oxidative stress are upregulated in aged rodent brains [277]. In humans, chronic hypoperfusion can additionally augment inflammasome signaling [278]. Unfortunately, the available molecules that target inflammasomes (anakinra, rilonacept, and canakinumab) have poor BBB penetrance and have not been tested yet in neurodegenerative diseases [258]. Sulphoraphane, a natural phytochemical also able to diminish inflammasome activation, has a similar poor bioavailability [279].

### 3.7. The Contribution of Necroptosis to Age-Related Neuroinflammation

Necroptosis is a programmed cell death pathway initiated when necroptotic stimuli, such as TNF-α, mTOR (mammalian target of rapamycin)/Akt activation, or oxidative stress phosphorylate, activate receptor-interacting protein kinase 1 (RIPK1) and RIPK3 and, in turn, phosphorylate mixed lineage kinase domain-like (MLKL) protein. Following phosphorylation, MLKL oligomerizes and binds to the cell membrane, which becomes permeabilized and releases cellular components that can exacerbate inflammation [280]. Necroptosis appears to increase in the CNS in aging, as well as in several neurodegenerative and inflammatory diseases, as suggested by the increased expression of RIPK1, RIPK3, or MLKL [281]. However, the increased expression of phosphorylated MLKL is not evenly distributed across the brain, occurring mainly in the hippocampus and fifth cortical layer for yet-unknown reasons. It may be that hippocampal neurons are more vulnerable to TNF-α-induced neuronal necroptosis, and blocking necroptosis in RIPK3 knockout mice significantly reduced neuronal loss after an intracerebroventricular injection of TNF-α [282]. Aside from neurons, a small percentage of microglia also show an increased expression of phosphorylated MLKL in the brains of aged mice [281].

## 4. Triggers of Neuroinflammation and Inflammaging

### 4.1. The Bidirectional Relationship between Circadian Rhythm Dysfunction and Aging

Most organisms have circadian clocks that ensure physiological and behavioral adaptation to the 24-h light–dark cycle of Earth [283]. In mammals, the “master clock” is situated in the suprachiasmatic nucleus of the hypothalamus, which receives input from the retina entraining the cellular clocks of neurons to the external light–dark cycle [284] and regulating the endocrine and autonomic nervous system function [285]. Each cell has a molecular clock formed by a positive transcriptional limb composed of the transcription factor BMAL1, which forms heterodimers with CLOCK or NPAS and binds to E-box motifs to drive circadian transcription, and a negative limb consisting of the PERIOD and CRYPTOMCHROME families of proteins, which inhibit BMAL1 function [286]. A series of secondary clock proteins tune the core clock to a 24-h period by regulating 10–50% of all transcripts in a cell [283,287].

Aging is associated with a series of sleep changes, including difficulty falling and staying asleep, increased sleep-to-wake transitions [288], or increased daytime napping, with a decrease in the slow-wave sleep important for protein clearance [289] and the consolidation of memory [290]. Melatonin, a pineal gland hormone that normally induces sleep (possibly by acting on BMAL1) [291], has been inconsistently shown to decrease with age, which, together with the declining expression of melatonin receptors, may lead to alterations in the sleep–wake cycle during advanced age [292]. Glucocorticoid secretion is also regulated via the suprachiasmatic nucleus, and it contributes to synchronizing the peripheral molecular clocks [283]. Aging is associated with alterations in the rhythmical variations of circulating cortisol [293], while impairments in hippocampal glucocorticoid signaling may be involved in the depletion of neural stem cells [294].

A series of morphologic and functional changes have been described in the suprachiasmatic nucleus with aging, such as a loss of GABAergic synapses, a decrease in the expression of neuropeptides (arginine, vasopressin, and vasoactive intestinal peptide), and impairments in the rhythm of neuronal firing [283]. Some researchers have also documented an altered expression rhythm of genes such as *Bmal1*, *Clock*, or *Per2* [295], possibly related to the accumulation of senescent cells in the CNS and suprachiasmatic nucleus [283]. Glial cells, such as astrocytes and microglia, also include molecular clocks with important functions in entraining activity rhythms [296] and regulating BBB permeability [297].

Both in vitro and animal studies have convincingly demonstrated the circadian variation in the strength of immune responses at the periphery [298] and in the CNS [299]. For instance, light-induced circadian-rhythm disruption exacerbates the release of TNF-α and IL-6 in response to a lipopolysaccharide insult, while the latter differentially elicits the immune response, depending on the time of day [300]. One may assume that a more active immune system during the transition from resting to waking is an attempt to prepare the body for exposure to pathogens during the day and to minimize energy expenditure induced via the immune system [283].

Brain-specific *Bmal1* knockout, as well as *Clock/Npas2* double knockout, is associated in mice with age-dependent increased oxidative stress and chronic inflammation [301], while *Bmal1* knockout in monkeys leads to immune activation and depressive symptoms [302]. The expression of microglial *Bmal1*, *Per2*, and *Nr1d1* is regulated via the molecular clock [303], and the levels of pro-inflammatory cytokines TNF-α, IL-6, or components of the NLRP3 inflammasome, show circadian variations as well [304], which may be abolished by aging [305]. Moreover, BDNF and the activation of the Nrf-2-dependent pathway provide protection against oxidative stress, while NF-κB-dependent inflammation is regulated via the astrocytic molecular clock [303].

### 4.2. The Role of the Gut Microbiota

Increasing evidence suggests that gut microbiota impact brain functions and are involved in the pathophysiology of neurodegenerative diseases [186]. The human digestive tract contains 10^13^–10^14^ living microorganisms which contribute to nutrient absorption and vitamin synthesis. Host immunity prevents extreme pathogenic changes in the gut microbiota, but aging and dietary habits (including modifications of diet due to age-related enzyme deficiencies, chronic constipation, and the excess use of laxatives) significantly change the gut microbiota towards a more pro-inflammatory composition, increasingly linked to somatic decline.

At the family level, *Bacteroidaceae*, *Ruminococcaceae,* and *Lachnospiraceae* decrease with aging, while *Christensenellaceae* and *Synergistaceae* increase. As for genera, *Eggerthella*, *Akkermansia*, *Bilophila*, *Escherichia*, *Desulfovibrio,* and *Anaerotruncus* are more prevalent with age, and *Faecalibacterium*, *Prevotella,* and *Bacteroides* are reduced in the elderly [306]. The human gut virome also changes with increasing age, with studies showing a decrease in viral richness [307]. An unbalanced diet, rich in fats and carbohydrates, such as the Western diet, leads to gut dysbiosis, and it causes metabolic endotoxemia and increases in serum markers of inflammation [308], being linked to the etiology of diabetes, metabolic syndrome, and cardiovascular diseases [309].

The gut is composed of epithelial cells connected via tight junctions, covered by a mucus layer with antimicrobial peptides and immunoglobulins A on the luminal side, and immune cells in the lamina propria [310]. Specialized epithelial cells (M cells) covering the dome of Peyer’s patches perform the immune surveillance of intestinal antigens transported to the lymphoid follicles [311]. In aging, the mucus layer undergoes thinning, the components of the tight junctions connecting epithelial cells are attenuated, and serum zonulin, a marker of a “leaky” gut, increases [312]. All of these changes suggest a weakening of the intestinal barrier. However, D’Amato and coworkers could demonstrate impairments in learning and memory in young mice following microbiota transplants from age-matched donor mice in the absence of abnormal gut permeability or an increase in circulating cytokine levels [313].

Metabolites produced via the gut microbiota significantly impact the intestinal barrier and systemic inflammation. While microbial components and small molecules [314], short-chain fatty acids, bile acids [315], methane, and hydrogen gas [316] exert anti-inflammatory effects, lipopolysaccharides, trimethylamine N-oxide, and ammonia may act as pro-inflammatory factors [315]. A “Leaky” intestinal barrier can be caused by the disintegration of the tight junctions connecting intestinal epithelial cells, induced through TNF via a pore pathway regulated via IL-13, or by loss of the intestinal epithelium caused by caspase-8-triggered apoptosis [317]. Gut inflammation then spreads via the lymphatic drainage pathway and systemic circulation following the disruption of the gut–vascular barrier [186].

Systemic inflammation weakens the BBB via several pathways. The integrity of the BBB relies on the Wnt/β-catenin, TGF-β, PDGF-β, and sonic hedgehog (Shh) signaling pathways [186]. Astrocytes are the main sources of Wnt signals in the neurovascular unit. The frizzled receptors of brain endothelial cells bind Wnt, leading to the sequestration of GSK-3β from β-catenin. However, GSK-3β activation via pro-inflammatory cytokines ignites the Akt/GSK-3β and weakens Wnt/β-catenin signaling. In the hippocampus of aged animals, an increase in TNF-α and NF-κB was associated with increased Akt/GSK-3β activity [318]. Astrocytes are also the main source of Shh, which is positively associated with tight junction expression [186]. IL-1β from peripheral circulation or released via activated microglia suppresses the astrocytic production of Shh, promotes the production of pro-inflammatory factors (such as CCL2) via astrocytes [319], and enhances endothelin-1 activity, which further downregulates Shh signaling and contributes to the uncoupling of the neurovascular unit and the weakening of the BBB [320]. IL-6 also reduces the expression of tight junctions, adherens junctions, claudin-5, or VE-cadherin in brain endothelial cells, while TNF-α decreases the thickness and stiffness of the glycocalyx [186]. Further, interferon-γ, IL-17A, and zonulin enhance the permeability of both the intestinal barrier and the BBB by modulating tight junctions and the associated cytoskeleton, opening the way for immune-cell transmigration into the CNS. Th17 cells can produce matrix metalloproteinases (MMP-3 and MMP-9) that further damage the BBB by decomposing the basal membrane [321]. Finally, activated microglia stimulate astrocytes to release TNF and glutamate and produce more chemokines to recruit leukocytes into the CNS [322].

### 4.3. Cholinergic Modulation of Neuroinflammation

Acetylcholine, one of the first identified neuromediators, acts on muscarinic (M1–M5) receptors, which are metabotropic G protein-coupled receptors, and nicotinic receptors (nAChRs), which are ionotropic cation channels [70], to exert its various functions in both peripheral and central nervous system. In the CNS, nAChRs are pentamers formed through combinations of α and β subunits, and they are expressed by neuronal and glial cells [323], including microglia and astrocytes.

The existence of a cholinergic anti-inflammatory pathway in the CNS was first proposed by Shytle in 2004, and this pathway is mediated via the activation of α7nAChRs [324]. The anti-inflammatory effect of α7nAChR stimulation in astrocytes is exerted via the activation of Nrf-2, which leads to the expression of a series of antioxidant genes and a decrease in the expression of p50, an inhibitor of IκB phosphorylation, with the subsequent inhibition of NF-κB nuclear translocation [325]. In microglia, α7nAChRs’ anti-inflammatory effect is also mediated via the activation of the Nfr-2 pathway, as well as the phosphorylation and activation of p38, p44/42, and c-Jun-N-terminal kinase (JNK) MAP kinases [70].

The vagus nerve’s visceral afferents in the gut can be stimulated via IL-1, the nerve conveying the information to the nucleus of the solitary tract, which, in turn, projects to the dorsal motor nucleus of the vagus from where efferent fibers reduce the formation of pro-inflammatory cytokines through splenic lymphocytes and macrophages via the α7nAChRs [326].

During aging, a slight decrease in both muscarinic and nicotinic AChRs, as well as a reduced formation of acetylcholine, has been documented [71], and it may underlie both cognitive decline and potentiate chronic neuroinflammation.

### 4.4. Glial Cells and Sex Differences in Brain Aging

Both sex chromosomes and gonadal hormones influence the modifications induced via aging in the nervous system [327], and women are at greater risk of developing dementia [328]. Moreover, the nervous system has gender-specific responses to physiological and pathological challenges related to sex differences in systemic immunity, metabolism, and cardiovascular function, which are also caused by hormones and sex chromosomes [329]. Due to the complex functions of glial cells in the brain, as well as their marked sexual dimorphism and interaction with gonadal hormones, glial cells are greatly responsible for these gender differences in brain aging [330].

Oligodendrocytes show higher densities in the corpus callosum, fornix, and ventral funiculus of the spinal cord in male mice and rats, with thicker myelin sheaths, differences that may be explained by the effect of androgens acting on specific receptors [331]. Transcriptomic analyses of oligodendrocyte precursor cells showed gender-related differences in the expression of genes encoding for proteins involved in myelination, proliferation, cell cycle, and maturation [332], which may underlie different functional properties: female oligodendrocyte-precursor cells have a greater migratory ability and are more proliferative, while male ones have higher differentiation and myelinating properties [332].

Female and male astrocytes show differences in the expression of genes in response to inflammatory insults and in the recruitment of immune cells [330]: in vitro, inflammation stimulates the phagocytosis of cellular debris via male astrocytes but inhibits phagocytosis in female ones [333]. Astrocytes also make important contributions to the neuroendocrine control of the metabolism, and they are differentially affected by metabolic challenges, which may explain the gender differences in the onset of obesity and related diseases that impact systemic inflammation and the CNS [334].

Microglia vary between sexes in terms of gene expression, number, and morphology [335], as well as the response to brain insults. For instance, female microglia transplanted into the brains of male mice subjected to focal cerebral ischemia improved outcomes, as opposed to male microglia transplanted into female animals [336]. In addition, female microglia exhibit an increased expression of inflammation-related factors [337] with advancing age, and they exhibit aging phenotypes earlier [338]. Aging microglia also respond differently to estradiol: in female microglia isolated from the adult hippocampus and stimulated with lipopolysaccharides, estradiol reduces IL-1β expression, as opposed to male microglia [339].

Moreover, genes situated on the sex chromosomes may contribute to generating gender differences in brain pathology. For example, extreme downregulation of the Y chromosome in humans increases the risk of Alzheimer’s disease [340].

Gonadal hormones exert their effect on the CNS through their steroid metabolites, which act on androgen, estrogen, and progesterone receptors expressed via glial cells [330]. The levels of these gonadal hormones decline with aging, leading to a changing regulation of glial cell function [341]. Tibolone, a synthetic steroid hormone used for the treatment of menopausal symptoms, stimulates the phagocytosis of cellular debris via astrocytes more significantly and acts on different receptors in women compared to men [333].

Still, much research is needed to elucidate the specific cellular and molecular mechanisms that underlie the sex-specific characteristics of nervous-system aging and disease and to develop gender-specific precision medicine. These gender-related differences in pathophysiology and the course of neurological diseases may also bias clinical trials. However, glial cells appear to represent a relevant target for sex-specific interventions against age-associated neurodegeneration [330].

## 5. Consequences of Inflammaging on the Brain

During life, there is a continuous duel between damage accumulation from environmental and endogenous events and resilience mechanisms that cope with stressors and resolve damage [342]. With increasing age, these resilience mechanisms become less effective and allow for the accumulation of molecular and cellular damage expressed as inflammaging, susceptibility to chronic diseases, physical and cognitive impairment, frailty, and death [343]. The aging of the immune system, meant to react to commensal and pathogenic microorganisms, pathogen-associated molecular patterns, and damage-associated molecular patterns from endogenous and exogenous sources, leads to a state of pro-inflammatory activation characterized by high circulating levels of pro-inflammatory cytokines and localized tissue inflammation, as well as an either blunted or excessive response to antigens and pathogens [343].

All the postulated hallmarks of aging are linked to inflammation via a bidirectional feedback loop:

- Genomic instability can lead to inflammation by activating poly (adenosine diphosphate-ribose) polymerase 1 (PARP1) via single-stranded DNA breaks. PARP1 activity causes NAD+ depletion, which interferes with SIRT activity. SIRT depletion causes the accumulation of mitochondrial DNA damage and mitochondrial dysfunction, as well as the activation of the NLRP3 inflammasome [343]. Damaged DNA can also leak into the cytoplasm, triggering the cGAS-STING pathway [344]. Conversely, chronic inflammation can induce genomic instability through elevated TNF-α levels binding to its receptors and phosphorylation of p47 phagocyte oxidase, followed by the recruitment and plasma membrane translocation of TNF receptor-associated factor 4, which facilitates nicotinamide adenine dinucleotide phosphate hydrogen oxidase activity and leads to ROS production [345], or via inducible nitric oxide synthase, which reduces DNA methyltransferase 1 and leads to DNA damage and hypomethylation [346].
- Telomere dysfunction, together with DNA damage, activate p53, which suppresses peroxisome proliferator-activated receptor gamma coactivator 1 alpha (PGC-1α) and mitochondrial sirtuins, leading to mitochondrial dysfunction and oxidative stress [347]. In addition, the downregulation of PGC-1α induces the NLRP3 inflammasome, which leads to caspase-1 activation and the conversion of pro-IL-18 to IL-18 [348]. Inflammatory cytokines, mainly TNF-α, downregulate telomerase activity and lead to accelerated telomere shortening [349], while type I interferons also inhibit telomerase activity and promote telomere erosion [350].
- Epigenetic alterations, such as DNA methylation and histone modifications, can lead to inflammatory consequences, activating genes involved in the inflammatory responses, such as NF-κB or interferons [350], while histone modifications activate or repress genes via histone acetyltransferases or histone deacetylases [351].
- Continuous exposure to mutagens leads to the accumulation of misfolded proteins that overwhelm the cellular proteostasis machinery and elicit the unfolded protein response through the activation of inositol-requiring transmembrane kinase/endoribonuclease 1α, the activating transcription factor 6, and the protein kinase R-like endoplasmic reticulum kinase (PERK), resulting in NF-κB activation [352]. Protein aggregates can be recognized as DAMPs by PRRs and cause the assembly of the NLRP3 inflammasome, as well as caspase-1 cleavage and increased IL-18 and IL-1β production [353]. On the other hand, systemic chronic inflammation alters both protein folding and degradation through PERK-dependent phosphorylation of eukaryotic translation initiation factor 2, thereby hindering the translation of misfolded proteins, and by decreasing the influx of translated proteins into the ER [354]. Moreover, increased levels of ROS as a consequence of inflammation impair protein folding and facilitate the formation of misfolded and cytotoxic protein aggregates [355].
- Impaired autophagy allows for the accumulation of protein aggregates, dysfunctional organelles, and cytosolic DNA, thereby contributing to inflammation signaling [356]. Caloric restriction or caloric restriction-mimetic therapies have been shown to activate autophagy and exert anti-inflammaging effect [350]. Chronic inflammation has been shown to impair autophagy mainly in neurodegenerative diseases such as AD, PD, Huntington’s disease, or amyotrophic lateral sclerosis, in which the increased levels of pro-inflammatory cytokines (IL-1β, IL-6, and TNF-α) correlate with decreased levels of autophagy markers (Beclin-1, p62, and LC3 II) [357].
- Nutrient sensing is regulated through the insulin and insulin-like growth factor 1 signaling pathways (known as the IIS pathways), the mTOR and AMPK pathways, and sirtuins [350]. Aging is associated with a decline in insulin clearance and insulin resistance, leading to dyslipidemia [358]. Triglycerides can induce NF-κB in macrophages, while free fatty acids and chronic hyperinsulinemia activate the mTOR pathway [359]. While the mTOR kinase senses amino acid levels and controls cell growth, the AMPK–sirtuin pathway senses nutrient scarcity [350]. Prolonged IIS signaling activates mTOR complex 1, resulting in the inhibition of IKβ and the overactivation of IKK [360]. Pro-inflammatory cytokines can interact with mTORC1, accelerate aging [361], and inhibit AMPK activity [362].
- The bidirectional relationship between mitochondrial dysfunction and inflammation has been subject to extensive research. The cytoplasmic release of mitochondrial DNA activates TLRs and ignites NF-κB signaling, inducing the expression of pro-inflammatory cytokine genes [363]. Cardiolipin stimulates the release of cytochrome c and leads to apoptosis [364]. Conversely, inflammation impairs mitochondrial function through inflammasome activation and by promoting excess ROS production, with increased oxidative damage of mitochondrial proteins, lipids, and DNA [365].
- Senescent cells secrete the SASP (consisting of a wide array of pro-inflammatory cytokines) and “spread” cellular senescence in a paracrine manner [366], while oxidative molecular and DNA damage inflicted via the oxidative stress associated with chronic inflammation can trigger cellular senescence [350].
- Intercellular signaling includes endocrine, neuronal, and neuroendocrine pathways, as well as the cell-to-cell exchange of vesicle-packed and free soluble factors, inflammatory signals included [16]. Inflammation at the level of the hypothalamic-pituitary axis contributes significantly to metabolic dysregulation [367] and decreased insulin sensitivity, leading to impaired immune cell function [350]. Extracellular vesicles are released via most cell types, and they contain proteins, lipids, and nucleotides, sharing them between the donor and recipient cell and thereby linking hallmarks of aging through inflammatory mediators [368].
- Multiple factors (DNA damage, telomere attrition, and epigenetic dysregulation) cause a decline in the proliferative and regenerative capacities of stem cells, leading to the accumulation of senescent cells [369]. In turn, senescent cells can exacerbate stem-cell exhaustion via the SASP cytokines that favor the infiltration of the tissues with immune cells, further impairing stem-cell function [370].

Although the brain has long been considered an immune-privileged organ with limited exposure to peripheral immune challenges, in the context of inflammaging and the dysregulation of the neuro-immune axis, the brain becomes increasingly vulnerable [343]. Aging is the most prominent risk factor for a series of neurodegenerative diseases such as AD, Parkinson’s disease, or other forms of dementia, and in all of these conditions, research has shown an increase in circulating inflammatory cytokines and chemokines [9,10]. There is an age-related increase in the permeability of the BBB due to the loss of tight junctions and a shift from tightly regulated receptor-mediated transcytosis toward nonspecific transcytosis of plasma proteins [191]. Moreover, circulating inflammatory factors, such as TNF-α, can further enhance BBB permeability by suppressing the expression of tight junctions. Circulating TNF-α, IL-1β, and IL-6 bind to receptors on endothelial cells and induce the expression of cellular adhesion proteins that further promote inflammation by triggering NF-κB signaling, by enabling the tethering of circulating myeloid cells to the brain endothelia, and by igniting microglial activation [371]. In addition, inflammatory cytokines and chemokines penetrate the BBB through active-transport or nonspecific caveolar transcytosis [191]. Chronic infections (for example, periodontal diseases), acute infections, inflammatory diseases (for example, rheumatoid arthritis and inflammatory bowel disease), or obesity (through the hypoxia of adipocytes, endoplasmic reticulum stress, the impairment of PPAR receptors, and the activation of inflammasomes and of TLRs) [372] can all increase the levels of the circulating inflammatory cytokines that eventually reach the brain through the “leaky” BBB. Many inflammatory mediators shown to increase with aging (IL-1β, IL-18, and sTNF-R1) are upregulated to an even greater extent in neurodegenerative conditions such as AD and PD [373,374]. Interestingly, many genes shown to be associated with late-onset sporadic AD are in or near genes important for immunity or expressed via brain immune cells [375].

Age itself is associated with phenotypic changes to microglia and astrocytes that are amplified by the glial inflammatory response after both central and peripheral immune challenges [376]. Microglial changes include a deramified morphology, increased cytokine and chemokine expression, and an upregulation of MHC-II and TLRs [377], while astrocytes exhibit elevated glial fibrillary acidic protein (GFAP) expression and hypertrophic morphology [343]. Aged glial cells respond less efficiently to antigens, including amyloid-β and α-synuclein, and they have reduced phagocytic and anti-inflammatory capacity [378]. Moreover, activated glial cells exert a neurotoxic effect, express increased levels of cytokines and chemokines, and release DAMPs that promote neuroinflammation via the multiple pathways discussed above.

Resting microglia perform crucial physiological functions in the regulation of neuronal activity, synaptic transmission, and the formation, modification, or elimination of synapses [379]. Under normal conditions, complement proteins specifically bind to apoptotic, immature, or weak developing synapses in the CNS, structures that are recognized by complement receptors and consequently engulfed. Another signal that promotes synaptic pruning is phosphatidylserine, which occurs on apoptotic or injured dendrites and is recognized by microglial TREM2 receptors [380]. In addition, microglia can regulate phagocytosis and synapse elimination through the interaction of the microglia-expressed fractalkine receptor CX3C motif chemokine receptor 1 (CX3CR1) with its ligand, CX3CL1, expressed by neurons [381]. Microglia are also critically involved in the modulation of synaptic plasticity, defined as the ability of the CNS to modify synapses and neural connections in response to synaptic activity and sensory and motor experiences. They also modulate long-term potentiation (LTP) and long-term depression (LTD) via the purinergic receptor P2RY12, PI3K/BDNF signaling, and fractalkine signaling, respectively [382]. However, a disrupted balance between pro-inflammatory and anti-inflammatory markers released via microglia significantly impairs synaptic plasticity [383]. Experimentally, a lipopolysaccharide challenge causes the microglial release of TNF-α (resulting in a transient increase in LTP) and IL-1β (which impairs LTP in CA1 neurons) [382]. Microglia can also modulate synaptic transmission through BDNF expression via PI3K/BDNF signaling [384]. As for adult neurogenesis, microglia from the subgranular zone and subventricular zone play an active role via the phagocytosis of apoptotic newborn neuronal cells [385], and they direct the migration of newly formed cells [386]. The wide range of inflammatory mediators released via primed and activated microglia (IL-6 and TNF-α) have been convincingly shown to reduce neurogenesis [387].

In addition, adaptive immune alterations significantly contribute to the progression of neurodegenerative diseases and persist throughout the disease. Self-antigens, such as amyloid β, could induce autoreactive effector T-cells that drive pro-inflammatory and neurodestructive cascades [388], acting together with an impaired function of the regulatory T-cells [389].

## 6. Identifying Inflammation in the CNS

### 6.1. Imaging Inflammation in the CNS

Molecular imaging of the inflammatory processes is essential in both clinical practice and research. The main molecular target in functional imaging of inflammation is translocator protein (TSPO), a 169-amino-acid protein on the outer mitochondrial membrane that binds to benzodiazepines [390] and is upregulated in inflammatory processes. Several radiolabels allow the assessment of TSPO expression via single-photon emission computerized tomography (SPECT) or positron-emission tomography (PET). Alternatively, several radiotracers can bind to activated microglia.

The first generation of tracers included Ro5-4864 and isoquinoline. Ro5-4864 is a member of the 4-chlorodiazepam family which, labeled with the C-11 isotope, can be used in the PET technique, binding to TSPO but not to the benzodiazepine subunit of the GABA receptor [391]. Isoquinoline (11-C-PK-1195) binds to activated microglial cells.

The second-generation tracers have higher binding specificity to TSPO and comprise phenoxy-phenylacetamide [11C]DAA1106 and [18F]DAA1106. They also bind to microglial cells, but their use is limited due to their binding to astrocytes and other immune cells [391,392].

Third-generation radiolabels are more specific in the imaging of activated microglia. This group includes tricyclic radiotracers such as [18F]GE180, [18F]FEBMP, or [11c]vinpocetine, as well as modified agents from the previous generations: [18F]FE-DAA1106, [18F]PBR28, [18F]PBR111, [11C]DPA-713, or [18F]DPA-714 [393,394].

### 6.2. Inflammatory Biomarkers

While CSF levels of beta amyloid (Aβ) and tau isoforms, together with markers of neuronal loss, such as neurofilament light (Nfl), are increasingly used as biomarkers in Alzheimer’s disease, neuroinflammatory markers are beginning to be evaluated to allow for the stratification of subjects according to the underlying molecular and cellular mechanisms leading to their impairments. Although it is difficult to capture the entire spectrum of the ongoing neuroinflammatory process through a single measurement of a panel of biomarkers, and the reported results are sometimes conflicting (probably varying with the disease stage and underlying comorbidities), serial measurements have started to reveal interesting associations, as shown in Table 3 below.

## 7. Therapeutic Approaches to Modulating Inflammaging

Several lifestyle approaches can delay aging and help maintain health [399]. A diet rich in certain metabolites, micronutrients, and phytochemicals contributes to epigenetic modulation and diminishes the risks of age-related diseases and inflammaging [400]. Consuming whole-grain cereals, vegetables, fish, and fruits, as in the Mediterranean diet, has been shown to have protective, antioxidant, and anti-inflammatory effects [401]. Furthermore, although not yet verified in humans, caloric restriction has been shown to prevent the onset of immunosenescence in animal models and extend lifespans [402]. Several plant supplements, such as polyphenols, curcumin, sulforaphane, or quercetin, have proven antioxidant and anti-inflammatory activities [279], acting via many pathways, despite their limited ability to cross the BBB.

Physical exercise leads to the release of a series of humoral factors from skeletal muscles, liver, and adipose tissue, which mediate several beneficial effects. One such factor is IGF1, which crosses the BBB via receptor-mediated transport and promotes synaptic plasticity and neurogenesis in the CNS [403]. Moreover, exercise prevents DNA damage, promotes telomerase activity [404], and reduces oxidative stress [405].

Psychological stress continuously activates the hypothalamus–pituitary–adrenal axis and causes persistent elevations of glucocorticoid levels, which lead to hippocampal atrophy [406]. As such, maintaining a good mental state can also delay aging [407].

Plant-derived drugs, such as flavonoids and icariin (a natural flavanol glycoside) enhance SIRT6 enzyme expression and repress NF-κB inflammatory signaling pathways [408]. Metformin inhibits the mTOR pathway, activates AMPK, and reduces ROS levels, insulin, and insulin-like growth factor-1 signaling [409].

Maintaining a healthy gut microbiota and the use of probiotics have been shown to reduce the level of systemic inflammatory factors and the expression of aging markers p53 and p16 [410] and to reduce sarcopenia caused by prolonged immobilization [411].

When referring strictly to neuroinflammation and neurodegenerative diseases (mainly AD), epidemiological studies have suggested that the long-term use of non-steroidal anti-inflammatory drugs (NSAIDs) was linked to a decreased risk of AD [412] and PD [413]. A follow-up analysis of the ADAPT clinical trial showed a modest effect of naproxen in preventing AD [414]. A novel non-steroidal anti-inflammatory drug (CHF5074 or itanapraced) lacking cyclooxygenase inhibitory activity was shown to restore normal microglial function, increase phagocytosis, and decrease the production of pro-inflammatory cytokines [415], and it has completed several phase 2 clinical trials [9]. Monoclonal antibodies against TNF-α are already used for autoimmune and inflammatory diseases, and they have also been evaluated in AD. The selectively soluble TNF inhibitor XPro-1595 inhibits TNF receptors type 1 and has been shown to reduce Aβ plaques, restore long-term potentiation, and prevent synaptic loss in mice [416]. It has completed a phase 1 open-label safety and tolerability study (NCT03943264) with unpublished results. The tyrosine kinase inhibitor dasatinib, used with quercetin, is currently in phase 1 and ½ clinical trials [9]. Inhibitors of p38 MAP kinase (neflamapimod and MW150) have also entered clinical trials [417]. Monoclonal antibodies targeting various receptors to interrupt several pathogenic neuroinflammatory cascades are clinically tested in phase 1 and 2 trials, including AL002 targeting TREM2 receptors, AL003 directed against CD33, daratumumab, a humanized IgG1κ monoclonal antibody targeting the CD38 epitope, or canakinumab, developed for use against IL-1β [9]. Several other anti-inflammatory therapeutic strategies are being evaluated in preclinical stages.

Finally, senotherapies are in active research. By secreting pro-inflammatory cytokines, senescent brain cells are a source of damaged macromolecules that propagate a pro-inflammatory response in the brain microenvironment and create harmful conditions with a significant negative impact on brain functioning. Eliminating senescent cells with senolytic compounds, or shaping the SASP with senomorphics, are exciting new strategies still in very early infancy. Most research is in preclinical stages, but they could help us achieve healthy aging and avoid the cognitive dysfunction, sarcopenia, and physical frailty associated with aging. Table 4 summarizes these attempts to modulate brain aging and neuroinflammation.

## 8. Challenges and Future Directions

Although the involvement of neuroinflammation in the pathophysiology of neurodegenerative diseases and aging of the CNS has been consistently shown in research, and the link between chronic peripheral inflammation and neuroinflammation has been convincingly demonstrated, we are still far from fully explaining the mechanisms underlying these links. A series of complex interactions between genetics, environment, and lifestyle further shape our immune system and modulate the aging process.

Research using the classical in vitro and in vivo models is ongoing.

- In vitro models consist of the following:
  - Replicative senescence models in which hydrogen peroxide is used to cause stress-induced premature senescence [420].
  - Oncogene-induced senescence, achieved via the activation of various oncogenes [421].
  - Chemotherapy-induced senescence, usually using doxorubicin [422].
- In vivo models include the following:
  - Induced aging mouse models, with the preferred agent being D-galactose [423].
  - Genetic mouse models, using particular senescence-accelerated mouse/prone (SAMP) strains, such as the SAMP8 strain [424].
  - Premature aging models based on the identification of the genetic mutations causing human progeroid syndromes and genetically modifying rodents accordingly [425].
  - Studying animals with naturally long lifespans or human centenarians.

In the future, spatial single-cell technologies (spatial transcriptome and spatial metabolome) will allow the construction of three-dimensional aging atlases at the organ level [426]. However, these models are less sensitive in identifying the interactions of specific brain regions with each other, a problem that might be solved using brain organoids [427]. Finding ways to vascularize these brain organoids and complement them with microglial cells could significantly advance our knowledge of neuroinflammation and brain aging [428].

In our view, before the much-awaited future innovative therapeutic strategies can enter clinical trials, several key areas require further investigation:

1. The identification of non-invasive biomarkers of neuroinflammation and immune dysregulation could be helpful in the early detection of impaired immune response, the prognostication of neurodegeneration, and the monitoring of treatment responses.
2. It is very likely that each stage of neuroinflammation will require targeted strategies for efficient modulation. Moreover, treating neurodegenerative diseases will need to combine immunomodulatory therapies with strategies aiming at clearing specific protein aggregates. Aside from exploring novel therapeutic targets, improving existing immunomodulatory therapies, with new drug-delivery systems, could enhance efficacy and minimize the side effects and off-target effects of drugs.
3. Given the influence of genetic factors and gender on the risk and progression of aging and neurodegenerative diseases, tailoring therapeutic interventions to individual patient profiles holds promise, and precision medicine strategies will have to incorporate computational modeling, algorithms, and multi-omics data integration to improve treatment selection.
4. Collaborative efforts between basic scientists, industry partners, and clinicians could accelerate the development of effective treatments against neurodegenerative and neuroimmune disorders.

A better understanding of the mechanisms linking all age-associated dysfunctions could open the perspective on ensuring healthy aging, retaining memories and physical fitness, the ability to make decisions, and all of those things that make long lives worth living.

## Figures and Tables

**Table 1:** Cytokines and chemokines expressed by senescent versus reactive astrocytes.

Astrocytic Phenotype	Cytokines	Chemokines	Ref.
Senescent astrocytes	IL-1α, Il-1β, Il-2, IL-6, IL-8, IL-10, TNF-α	GRO-α, IP10, RANTES, MIP-1α, MIP-2	[101,102,103]
Reactive astrocytes	IL-1α, IL-1β, TNF-α, IL-6, IL-8, IL-10, IL-12, IFN-α, IFN-β, IFN-γ	IP10, RANTES, CCL9, CCL10, CCL12, CXCL1, CXCL5, CXCL13, CXCL16, MIP-1α, MCP-1	[80,87]

Abbreviations: GRO-α—growth-regulated protein alpha (CXCL1); RANTES—regulated on activation, normal T-cell-expressed and -secreted (also known as CCL5); IP10—Interferon gamma-induced protein 10 (also known as CXCL10); MIP-1α—macrophage inflammatory protein-1α; MIP2—macrophage inflammatory protein 2; TNF-α—tumor necrosis factor α; MCP-1—monocyte chemotactic protein-1; IFN—interferon.

**Table 2:** Protective and detrimental microglial signaling pathways.

	Mediated by	Function	Effect on			Ref.
			Microglial Activation	Neuroinflammation	Neurodegeneration	
Protective microglial signaling pathways	TGF-β	Promotes a quiescent state of microglia, neuroprotective	Inhibits	Inhibits	Inhibits	[143]
	BDNF	Neurotrophic	No action	Decreases	Inhibits	[144,145]
	GDNF	Inhibits microglial activation	Decreases	Decreases	Decreases	[146]
	CD200 interacting with CD200R	Maintains microglia in a quiescent state	Decreases	Decreases	Decreases	[147]
	CXCL4/CCR5	Inhibits microglial activation	Decreases	Decreases	Decreases	[148]
	TNF-TNFR2	Neuroprotective and anti-inflammatory	Increases anti- inflammatory cytokines	Decreases	Inhibits	[149]
Detrimental microglial signaling pathways	TNF-TNFR1	Induces microglial activation	Increases	Increases	Promotes	[150]
	Astrocytic IL-33	Pro-inflammatory	Increases chemokine expression	Increases recruitment and infiltration of macrophages in the CNS	Increases	[151]
	TLRs	Pro-inflammatory	Increase in cytokine production	Increases	Promotes	[152]
	TREM2 receptors	Promote phagocytosis	Increase phagocytic activity	Increase	Increase	[153]
	CCL2, CCL21, CXCL10	Pro-inflammatory	Activate microglia	Promote recruitment of infiltrating immune cells	Promote	[121]

**Table 3:** Inflammatory biomarkers evaluated in cognitive impairment.

Marker	Sample	Correlations	Ref.
sTyro3	CSF	Old age, high tau levels, less of an Aβ burden Higher gray matter and white matter volume, less white matter hyperintensities, better memory, slower atrophy rates	[395]
sAXL	CSF	Old age, high tau levels, less of an Aβ burden Higher gray matter and white matter volume, less white matter hyperintensities, better memory, slower atrophy rates	[395]
	serum	Negatively correlated with structural imaging and cognitive function	[396]
sTREM2	CSF	Old age, high tau levels, less of an Aβ burden Higher gray matter and white matter volume, less white matter hyperintensities, better memory, slower atrophy rates	[395]
YKL-40	CSF	Old age, high tau levels, less of an Aβ burden Higher gray matter and white matter volume, less white matter hyperintensities, better memory, slower atrophy rates	[395]
	Serum	Correlated with structural changes but not with cognitive outcomes	[396]
IL-6	Serum	Negative correlation with structural measures of Braak regions	[396]
CRP	CSF	Male gender, high body mass index, greater vascular risk, less gray matter and white matter volume, worse memory	[395,397]
IL-18	CSF	Male gender, high body mass index, greater vascular risk, less gray matter and white matter volume, worse memory	[395]
Complement C4	CSF	Male gender, high body mass index, greater vascular risk, less gray matter and white matter volume, worse memory	[395]
C1q	CSF	Old age, high tau levels, less Aβ burden Higher gray matter and white matter volume, less white matter hyperintensities, better memory, slower atrophy rates	[395]
IP-10, sICAM-1	CSF	Correlated with neuropsychiatric symptoms	[397]
MIP-1, VEGF	Serum	Associated with neuropsychiatric symptoms	[397]
IL-12	Plasma	Associated with slower cognitive decline	[398]

Explanation: Tyro3 and AXL are type I receptor–tyrosine kinases that contribute to the clearance of apoptotic cells. YKL-40 is a chitinase-3-like protein 1 expressed via various cells, including macrophages, and used as a marker of disease activity in inflammatory and autoimmune diseases. Abbreviations: CRP—C reactive protein; MIP-1—macrophage inflammatory protein-1; IP-10—interferon-γ-inducible protein 10; IL—interleukin; sTREM2—soluble triggering receptor expressed on myeloid cells-2; sICAM-1—soluble intercellular adhesion molecule-1; VEGF—vascular endothelial growth factor.

**Table 4:** Therapeutic attempts to modulate brain aging.

Category	Intervention	Mechanisms of Action	Comments	Ref.
Lifestyle approaches	A healthy diet (whole-grain cereals, vegetables, fruits, and fish, supplemented with polyphenols, quercetin, curcumin, and sulforaphane)	Pleiotropic (antioxidant, anti-inflammatory actions)	Contributes to epigenetic modulation	[279,401]
	Caloric restriction	Increases SIRT activity	Not verified in humans but shown to extend lifespan in animals	[402]
	Physical exercise	Released exerkines promote neuroplasticity and neurogenesis, prevent DNA damage, promote telomerase activity		[403,404,405]
	Avoidance of stress	Dampens the hypothalamo–pituitary–adrenal axis		[407]
	Probiotics	Reduce systemic inflammatory markers, promote healthy gut microbiome		[410]
Strategies targeting neuroinflammation	NSAIDs	Reduce systemic inflammation and neuroinflammation	Epidemiologic studies have shown them to be efficient in preventing AD and PD	[412,414]
	Itanapraced (CHF5074)	Restores normal microglial function, decreases pro-inflammatory cytokine production	Completed phase 2 trials (NCT01303744, NCT01602393, NCT01421056)	[9,415]
	XPro-1595	Inhibits TNF receptors	Completed a phase 1 study (NCT03943264)	[416]
	Dasatinib	Tyrosine kinase inhibitor	Currently in phase 1 and ½ trials (together with quercetin—NCT04063124, NCT04785300, NCT05422885, NCT04685590)	[9,418]
	Neflamapimod and MW150	Inhibitors of p38MAPK	Completed 3 phase 2 studies (NCT02423200, NCT02423122, NCT03402659)	[417,418]
	AL002	Monoclonal antibody against TREM2 receptors	Evaluated in a phase 2 trial—NCT04592874	[9,418]
	AL003	Monoclonal antibody against the CD33 epitope	Evaluated in a phase 1 trial (NCT03822208)	[9,418]
	Daratumumab	Monoclonal antibody against the CD38 epitope	Currently tested in a phase 2 trial—NCT04070378	[9,418]
	Canakinumab	Monoclonal antibody against IL-1β	Assessed in a phase 2 trial—NCT04795466	[9,418]
Strategies targeting senescent cells	Senolytics and senomorphics	Eliminate senescent cells or modulate the SASP	Mostly in preclinical trials	[419]

## Data Availability

No new data were created.

## List of Abbreviations

AChRs	acetylcholine receptors
AD	Alzheimer’s disease
Akt	protein kinase B
ASC	apoptosis-associated speck-like protein
ATM	ataxia-teleangiectasia, mutated
ATP	adenosine triphosphate
ATR	ATM and Rad3-related
AQP4	aquaporin 4
BBB	blood–brain barrier
BDNF	brain-derived neurotrophic factor
BMAL1	Basic Helix–Loop-Helix ARNT Like 1
CARD	caspase activation and recruitment domain
CDK	cyclin-dependent kinase
cGAS	2′3′-cyclic GMP-AMP synthase
CNS	central nervous system
COX	cyclooxygenase
CSF	cerebrospinal fluid
DAMPs	danger-associated molecular patterns
DDR	DNA damage response
DNA	deoxyribonucleic acid
EAAT	excitatory amino acid transporter
Ecrg4	esophageal cancer-related gene 4
ER	endoplasmic reticulum
ERK	extracellular-regulated kinase
FADD	FAS-associated death domain
GABA	gamma-aminobutyric acid
GFAP	glial fibrillary acidic protein
GSK3β	glycogen synthase kinase-3 beta
HMGB	high-mobility group B
HSP	heat shock protein
ICAM-1	intercellular adhesion molecule-1
IFN	interferon
IIS pathways	insulin and insulin-like growth factor 1-signaling pathways
IκB	inhibitor of κB
IKK	IκB kinase
IL	interleukin
IRF3	interferon regulatory factor 3
JAK/STAT pathway	Janus kinase/signal transducer and activator of transcription pathway
JNK	c-Jun N-terminal kinase
Kir	inwardly rectifying potassium channels
LINGO-1	leucine-rich repeat and Ig-like domain-containing Nogo receptor-interacting protein 1
LRP-1	low-density lipoprotein receptor-related protein-1
MAPK	mitogen-activated protein kinase
MBP	myelin basic protein
MCP	monocyte chemoattractant protein
MDM2	mouse double minute 2
MHC	major histocompatibility complex
MIP	macrophage inflammatory protein
MLKL	mixed lineage kinase domain-like
MMP	matrix metalloproteinase
MRI	magnetic resonance imaging
mTOR	mammalian target of rapamycin
mTORC	mechanistic target of rapamycin complex
MyD88	myeloid differentiating factor 88
NADPH	reduced nicotinamide adenine dinucleotide phosphate
NF-κB	nuclear factor κB
NLRS	NOD-like receptors
NMDA	N-methyl-D-aspartate
NOD	nucleotide-binding oligomerization domain
NPCs	neural progenitor cells
Nrf-2	nuclear factor erythroid 2-related factor 2
NVU	neurovascular unit
OPCs	oligodendrocyte progenitor cells
PAMPs	pathogen-associated molecular patterns
PARP1	poly (adenosine diphosphate-ribose) polymerase 1
PD	Parkinson’s disease
PDGF	Platelet-derived growth factor
PERK	protein kinase R-like endoplasmic reticulum kinase
PET	positron emission tomography
PGC-1α	peroxisome proliferator-activated receptor gamma coactivator 1 alpha
PI3K	phosphoinositide 3 kinase
PPAR	peroxisome proliferator-activated receptor
PRRs	pattern-recognition receptors
RAGE	receptor for advanced glycation end product
RANTES	regulated on activation, normal T-cell-expressed and -secreted (also known as CCL5)
RB	retinoblastoma protein
RIPK	receptor-interacting protein kinase
RNA	ribonucleic acid
ROS	reactive oxygen species
RUNX1	runt-related transcription factor 1
SA-β-gal	senescence-associated β galactosidase
SALL1	Sal-like protein 1
SAMD	senescence-associated mitochondrial dysfunction
SASP	senescence-associated secretory phenotype
SIPS	stress-induced premature senescence
STING	stimulator of interferon genes
TBK1	TANK binding kinase 1
TER	telomerase RNA
TERT	telomerase reverse transcriptase
TFAM	mitochondrial transcription factor A
TGF	transforming growth factor
TIMP-1	tissue inhibitor of metalloproteinases 1
TJ	tight junction
TLRs	toll-like receptors
TNF-α	tumor necrosis factor α
TNFR	tumor necrosis factor receptor
TRADD	TNF-receptor-associated death domain
TREMs	triggering receptor expressed on myeloid cells
VCAM-1	vascular cell adhesion molecule 1
VEGF	vascular endothelial growth factor
